# Morphospecies and molecular diversity of ‘lace corals’: the genus *Reteporella* (Bryozoa: Cheilostomatida) in the central North Atlantic Azores Archipelago

**DOI:** 10.1186/s12862-022-02080-z

**Published:** 2022-11-04

**Authors:** Lara Baptista, Björn Berning, Manuel Curto, Andrea Waeschenbach, Harald Meimberg, António M. Santos, Sérgio P. Ávila

**Affiliations:** 1 Centro de Investigação em Biodiversidade e Recursos Genéticos, CIBIO, InBIO Laboratório Associado, 9501-801 Pólo dos Açores, Ponta Delgada, Açores, Portugal; 2 BIOPOLIS Program in Genomics, Biodiversity and Land Planning, CIBIO, Campus de Vairão, 4485-661 Vairão, Portugal; 3 MPB-Marine Palaeontology and Biogeography Lab, Universidade dos Açores, 9501-801 Ponta Delgada, Açores, Portugal; 4 Faculdade de Ciências da Universidade do Porto, Rua do Campo Alegre 1021/1055, 4169-007 Porto, Portugal; 5 University of Natural Resources and Life Sciences (BOKU), Department of Integrative Biology and Biodiversity Research, Institute for Integrative Nature Conservation Research, Vienna, Austria; 6 Oberösterreichische Landes-Kultur GmbH, Geowissenschaftliche Sammlungen, 4060 Leonding, Austria; 7 MARE, Marine and Environmental Sciences Centre, Faculdade de Ciências, Universidade de Lisboa, Campo Grande, 1749-016 Lisboa, Portugal; 8 Natural History Museum, London, UK; 9 Centro de Investigação em Biodiversidade e Recursos Genéticos, CIBIO, InBIO Laboratório Associado, Universidade do Porto, Campus de Vairão, 4485-661 Vairão, Portugal; 10 Departamento de Biologia, Faculdade de Ciências e Tecnologia, Universidade dos Açores, 9501-801 Ponta Delgada, Açores, Portugal

**Keywords:** Biodiversity, Biogeography, Integrative taxonomy, Oceanic islands, Phylogeny

## Abstract

**Background:**

As in most bryozoans, taxonomy and systematics of species in the genus *Reteporella* Busk, 1884 (family Phidoloporidae) has hitherto almost exclusively been based on morphological characters. From the central North Atlantic Azores Archipelago, nine *Reteporella* species have historically been reported, none of which have as yet been revised. Aiming to characterise the diversity and biogeographic distribution of Azorean *Reteporella* species, phylogenetic reconstructions were conducted on a dataset of 103 Azorean *Reteporella* specimens, based on the markers cytochrome *C* oxidase subunit 1, small and large ribosomal RNA subunits. Morphological identification was based on scanning electron microscopy and complemented the molecular inferences.

**Results:**

Our results reveal two genetically distinct Azorean *Reteporella* clades, paraphyletic to eastern Atlantic and Mediterranean taxa. Moreover, an overall concordance between morphological and molecular species can be shown, and the actual bryozoan diversity in the Azores is greater than previously acknowledged as the dataset comprises three historically reported species and four putative new taxa, all of which are likely to be endemic. The inclusion of Mediterranean *Reteporella* specimens also revealed new species in the Adriatic and Ligurian Sea, whilst the inclusion of additional phidoloporid taxa hints at the non-monophyly of the genus *Reteporella*.

**Conclusion:**

Being the first detailed genetic study on the genus *Reteporella*, the high divergence levels inferred within the genus *Reteporella* and family Phidoloporidae calls for the need of further revision. Nevertheless, the overall concordance between morphospecies and COI data suggest the potential adequacy of a 3% cut-off to distinguish *Reteporella* species. The discovery of new species in the remote Azores Archipelago as well as in the well-studied Mediterranean Sea indicates a general underestimation of bryozoan diversity. This study constitutes yet another example of the importance of integrative taxonomical approaches on understudied taxa, contributing to cataloguing genetic and morphological diversity.

**Supplementary Information:**

The online version contains supplementary material available at 10.1186/s12862-022-02080-z.

## Background

Bryozoans, although common components of marine benthic communities, remain some of the most understudied metazoans. As a result, detailed taxonomic studies combining morphological and molecular data are still missing for many higher taxa, even those that produce large colonies visible to the naked eye and occur at diving depths. The genus *Reteporella* Busk, 1884 is the most speciose and widespread of the 23 currently accepted genera within the cheilostomatid bryozoan family Phidoloporidae Gabb & Horn, 1862 [1, 2]. Like other phidoloporids, *Reteporella* occur in shallow coastal to upper bathyal benthic habitats throughout the world [3]. They often form lace-like, colourful, three-dimensional colonies, which earns them their common, yet misleading, name ‘lace corals’. Colony form can be influenced by environmental factors [4], enabling them to colonize a diversity of microhabitats [5]. Colony morphology in *Reteporella* is complex, typically displaying erect bilaminate, fenestrated sheets of zooids that form infolded, lobed or widely open calices. Taxonomic classification of *Reteporella* species has hitherto exclusively been based on morphological characters such as the shape of the peristome, morphology of the ovicell, and polymorphisms of the avicularia. However, taxonomic decisions are often hampered by the species’ complex morphology, which includes a pronounced ontogenetic gradient from the colony margin to its base, as well as the difficulty to distinguish interspecific differences from intraspecific variability in response to environmental conditions. This is particularly problematic if only fragmentary material is available for study [4, 6]. Molecular DNA analyses are therefore important to test the morphospecies taxon concept.

A recent large-scale phylogenetic analysis, which included three *Reteporella* representatives besides a number of other phidoloporid taxa [7], suggested that the genus is non-monophyletic, although the family is monophyletic. Precise knowledge of the ecology and (palaeo)biogeography of *Reteporella* species remains scarce, and even in comparatively well-studied regions, such as the North Atlantic and Mediterranean Sea, new taxa are regularly described (e.g. [8–12]). Studies on bryozoans from the central North Atlantic archipelago of the Azores peaked in the early 20^th^ century (e.g. [13–15]), while only few studies have made significant contributions to the knowledge of Azorean bryozoan diversity thereafter (e.g. [16, 17]). Recent times have seen a renewed interest regarding revisions of historical taxa in the Azores (e.g. [18–21]) and the identification of non-indigenous species (e.g. [22, 23]).

To date, five *Reteporella* species have been described from the Azores: *R. atlantica* (Busk, 1884), *R. gracilis* (Jullien, 1903), *R. oceanica* (Jullien, 1903), *R. rara* (Jullien, 1903), and *R. tristis* (Jullien, 1903). Another four species, which have their type localities outside the Azores [e.g. *Reteporella mediterranea* (Smitt, 1867) from the Mediterranean Sea, or *Reteporella sparteli* (Calvet, 1906) from the Gulf of Cádiz], were also reported to occur in the archipelago [13–16]. However, none of these species have as yet been revised using scanning electron microscopy (SEM) and their identity is unclear at present.

**Fig. 1:**
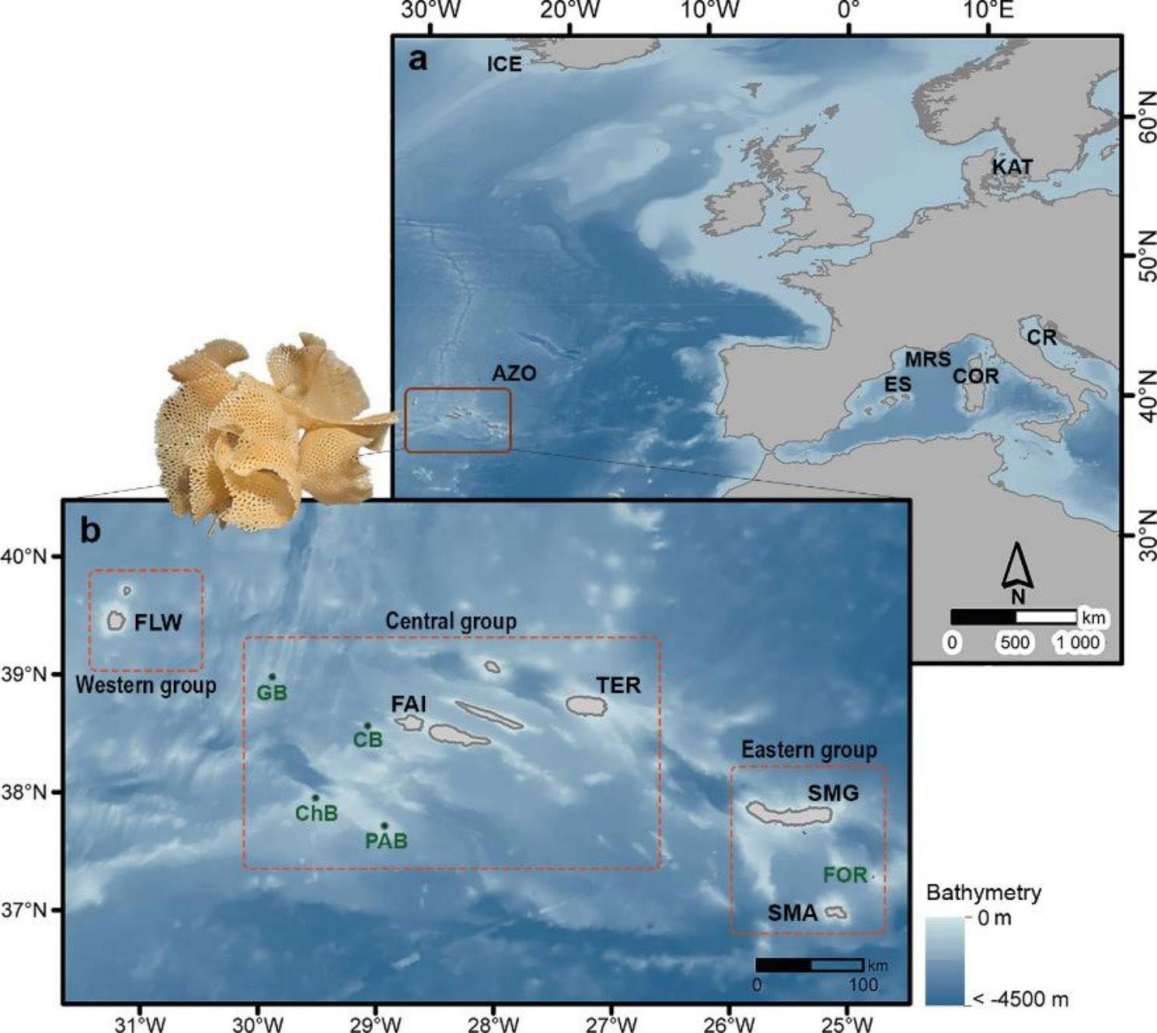
**Sampling localities in the North Atlantic Ocean and Mediterranean Sea. a** Overview of localities in the North Atlantic and the Mediterranean Sea; **b** Geographical locations of the Azorean islands (in black) and seamounts (in green). Island/seamount groups in the Azores (Eastern, Central, and Western groups) are delimited by dashed orange boxes. Coastline from the Portuguese Hydrographic Institute (https://www.hidrografico.pt/op/33) and bathymetry derived from GEBCO 2020 (https://www.gebco.net/data_and_products/gridded_bathymetry_data/). Populations labelled as follows: [AZO (Azores), ICE (Iceland), KAT (Kattegat), ES (Spain), MRS (Marseille), COR (Corsica), CR (Croatia), SMA (Santa Maria), SMG (São Miguel/Sabrina), TER (Terceira), FAI (Faial), FLW (Flores), FOR (Formigas), PAB (Princess Alice Bank), ChB (Chino Bank), CB (Condor Bank), GB (Gigante Bank)]

Ranging across the mid-ocean ridge, the Azores comprise nine oceanic volcanic islands divided into three distinct groups (Fig. 1). Spread over ~ 620 km along a WNW to ESE axis, the islands are located ~ 1,370 km W off the Iberian Peninsula, 840 km northwest of Madeira, ~ 1,500 km E off the nearest North American shelf (Newfoundland), and over ~ 3,800 km ENE off Cape Hatteras where the Gulf Stream is usually deflected to flow east towards the Azores. Given the prevailing set of sea-surface currents, the composition of the islands’ biota is peculiar and known as the “Azorean Biogeographical Paradox” [24, 25], one of the most puzzling conundrums in marine island biogeography. Despite the predominantly eastbound sea-surface circulation, most of the Azorean marine taxa show strong biogeographic affinities to the eastern Atlantic and the Mediterranean Sea [26–28, for details see 29]. Because of this mismatch, the patterns and processes of past colonization of the islands, and how connectivity is maintained with the surrounding mainland and archipelagos, are difficult to explain. This is particularly true for marine taxa with short-lived larvae such as the bryozoan genus *Reteporella*. With this study we aim to characterize the diversity and biogeographic distribution of *Reteporella* species in the Azores Archipelago, based on molecular and morphological datasets, as well as better understand the relationships with non-Azorean phidoloporids.

## Results

### Sequence data

**Fig. 2:**
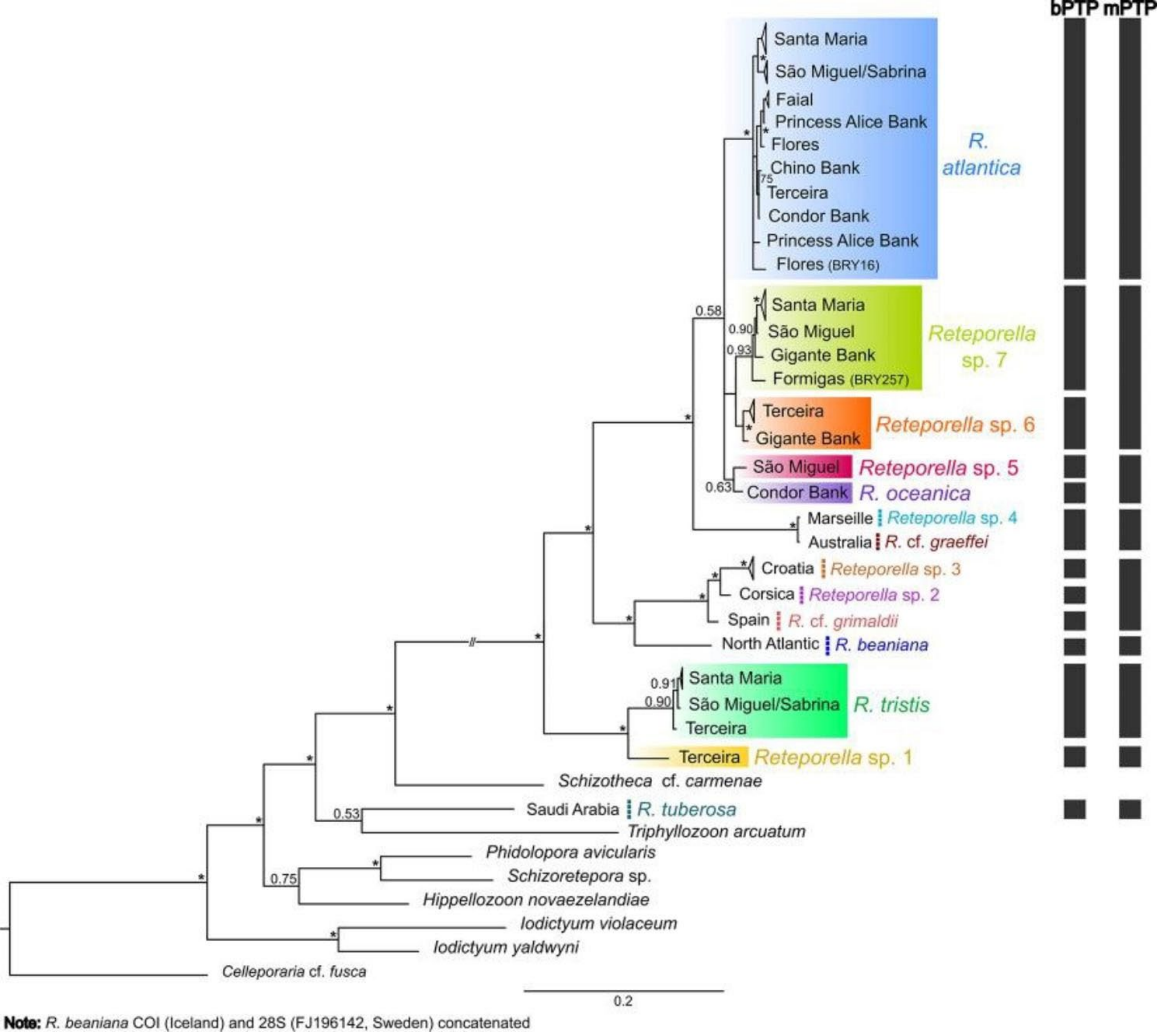
**Bayesian inference analysis of the concatenated dataset (COI + 16S + 28S)**. Constructed using MrBayes v3.2.7 software [30] under the following models of nucleotide evolution: GTR + I, F81 + I + G, GTR + G for the 1^st^, 2^nd^, and 3^rd^ codons of the COI, and GTR + I + G for both 16S and 28S markers. Values at the nodes correspond to posterior probability (PP); asterisk (*) indicates well-supported nodes (PP > = 95%). Known and putative new Azorean *Reteporella* species are indicated with different coloured shading. Non-Azorean *Reteporella* species are indicated by dashed coloured lines. Geographical origins of *Reteporella* samples are given as terminal labels. Other phidoloporids included in the analyses are identified with colour coding. The scale bar represents substitutions per site. *Reteporella* species boundaries inferred with bPTP and mPTP approaches, conducted on the single locus COI maximum-likelihood tree, are plotted on the right-hand side

**Fig. 3:**
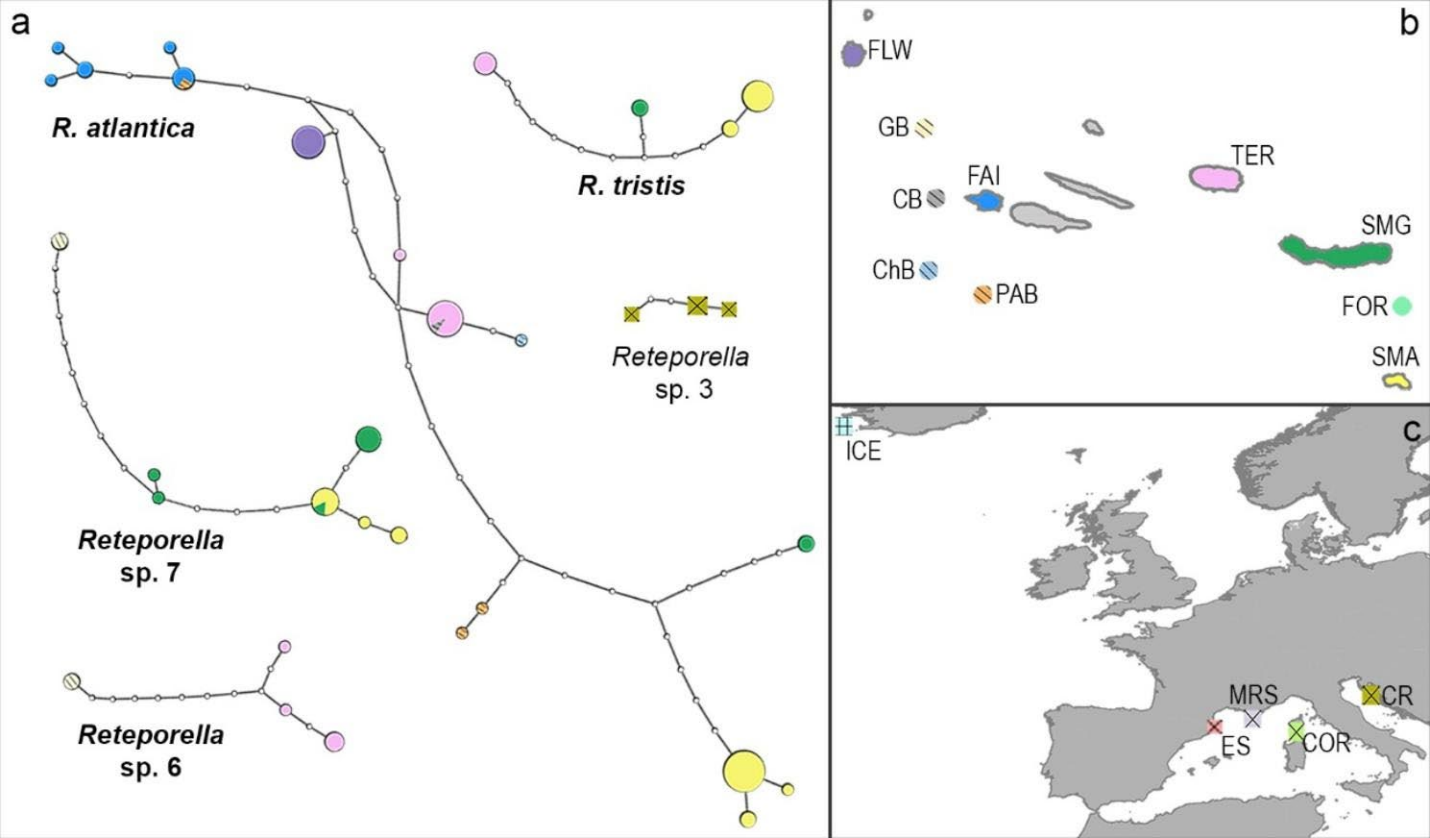
**COI haplotype network and geographic distribution of Reteporella haplotypes. a** Network reconstruction at 95% parsimony connection limit based on 97 COI sequences and 33 haplotypes, representing two known *Reteporella* species and three putative new species. Species represented by a single haplotype and/or individual [*R. oceanica*, *R. beaniana, Reteporella* spp. 1 and 5 (NE Atlantic), *R*. cf. *grimaldii*, *Reteporella* spp. 2 and 4 (Mediterranean), Reteporella cf. graeffei (Australia) and *R. tuberosa* (Saudi Arabia)] are not included in the dataset. Azorean haplotypes are represented by circles and taxon names in bold, whereas other Mediterranean haplotypes are represented by squares. Size of the circles and squares is proportional to the frequency of each haplotype; small uncoloured circles represent non-observed haplotypes; each line connecting haplotypes represents a single mutational change. Network obtained with TCS v1.21 [32] and tcsBU [33]; **b** Populations in the Azores are colour coded according to their geographical origin (map not to scale); **c** Populations in the North Atlantic and Mediterranean Sea are colour coded according to their origin. Populations labelled as follows: [SMA (Santa Maria), SMG (São Miguel/Sabrina), TER (Terceira), FAI (Faial), FLW (Flores), FOR (Formigas), PAB (Princess Alice Bank), ChB (Chino Bank), CB (Condor Bank), GB (Gigante Bank), ICE (Iceland), ES (Spain), MRS (Marseille), COR (Corsica), CR (Croatia)]

The following *Reteporella* sequences were obtained: 109 COI sequences, ranging from 552 to 575 bp, comprising 41 haplotypes; 69 16S sequences, ranging from 372 to 458 bp, comprising 21 haplotypes; 92 28S sequences 334 bp long, comprising 6 haplotypes. Lineage-specific mutations were detected in both the 16S and 28S data, including indels in the former. The delimitation of putative species was based on the comparison of results from the analyses of molecular data (phylogenies, haplotype network, and genetic distances). Morphological characters (see morphological analysis’ section below) allowed the validation of molecular lineages, identified as groups with COI genetic distances of over 3% and forming separated haplotype networks (indicated in Figs. 2 and 3).

The non-monophyly of the genus *Reteporella* is evident in the phylogenetic reconstructions, with *R. tuberosa* being more closely related to other phidoloporid genera, although its placement was variable or poorly supported (Fig. 2; Supplementary Material 2: Figs S1, S2). The concatenated phylogenetic reconstruction recovers the Azorean taxa as a paraphyletic assemblage to the inclusion of the Mediterranean, Australian, and North Atlantic taxa (Fig. 2). A clade composed of the Azorean taxa *R. tristis* (Jullien, 1903) and *Reteporella* sp. 1 forms the sister group to the remaining taxa. *Reteporella tristis* is represented by samples from Santa Maria and São Miguel islands, as well as Sabrina seamount (eastern group of islands) and Terceira (central group of islands). Its sister lineage, *Reteporella* sp. 1 from Terceira, which is distinguished from other *Reteporella* species by genetic distances ranging from 9.7 to 21% (COI), 2.6–20.8% (16S) and 0.3–1.5% (28S), is regarded as a putative new species (see also morphological analysis).

Among the remaining *Reteporella* taxa, the largest clade is composed of representatives of *R. atlantica* (Busk, 1884) that originated from all three island groups and four seamounts. Despite relatively low levels of genetic divergence within this group [2.1% (COI), 0.2% (16S), 0.3% (28S); Supplementary Material 2: Figs S1–3], samples from the easternmost islands of Santa Maria and São Miguel form a distinct clade amongst samples from more western localities (Fig. 2, Supplementary Material 2: Fig. S1). *Reteporella oceanica* (Jullien, 1903) from Condor Bank and *Reteporella* sp. 5 from São Miguel form a poorly-supported and well-supported clade in the concatenated (Fig. 2) and COI analyses (Supplementary Material 2: Fig. S1), respectively. These two taxa are part of a polytomy with *R. atlantica*, *Reteporella* sp. 7 from Santa Maria, São Miguel, Formigas (eastern island group) and Gigante Bank, and *Reteporella* sp. 6 from Terceira and Gigante Bank. The COI sequence divergence between *Reteporella* spp. 6 and 7 is 5.7–6.3%.

Among the non-Azorean *Reteporella* taxa (Fig. 2), a clade is recovered composed of the North Atlantic *R. beaniana* (King, 1846) and three Mediterranean taxa, from Spain (*R.* cf. *grimaldii*), Corsica (*Reteporella* sp. 2), and Croatia (*Reteporella* sp. 3). Genetic divergence between *R.* cf. *grimaldii* and *Reteporella* spp. 2 and 3 vary according to the marker [4.2–6.2% (COI), 0.4–0.8% (16S), and 0% (28S)]. The Australian *R.* cf. *graeffei* clusters with the Mediterranean *Reteporella* sp. 4 with near-zero branch lengths (but see Discussion), forming a sister clade to the larger Azorean clade composed of *R. atlantica*, *R. oceanica*, and *Reteporella* spp. 5–7. The interrelationships of the non-*Reteporella* phidoloporid taxa are mostly well-supported, except for the sister-group relationships between *R. tuberosa* and *Triphyllozoon arcuatum* (MacGillivray, 1889), and between *Hippellozoon novaezelandiae* (Waters, 1895) and (*Phidolopora avicularis* (MacGillivray, 1883) + *Schizoretepora* sp.).

Genetic divergence between the four large Azorean/European clades [(*Reteporella* sp. 1 + *R. tristis*), (*R. beaniana* + *R.* cf. *grimaldii* + *Reteporella* sp.2 + *Reteporella* sp. 3), (*R. atlantica + Reteporella* spp. 5–7)] is high in all the markers studied [maximum of 21% (COI), 10% (16S), 1.5% (28S)]. The maximum COI divergences among *Reteporella* species are of the same magnitude as those among other phidoloporid genera, ranging from 17% (*Schizoretepora* vs. *Phidolopora*) to 24.6% (*Schizoretepora* vs. *Iodictyum*). Among non-*Reteporella* genera, 16S divergences ranged from 5.5% (*Hippellozoon* vs. *Phidolopora*) to 13.2% (*Triphyllozoon* vs. *Iodictyum*). The 28S divergences ranged from 3.6% (*Triphyllozoon* vs. *Iodictyum*) to 7.3% (*Phidolopora* vs. *Iodictyum*) (Supplementary Material 2:Table S2).

The two tree-based species delimitation methods produced conflicting results, with bPTP inferring 13 *Reteporella* species and mPTP only 9 (Fig. 2). The mPTP analysis considers the following taxa as one species: *Reteporella* sp. 6 + sp. 7; *R. oceanica* + *Reteporella* sp. 5; *Reteporella* sp. 4 + *R.* cf. *graeffei; R.* cf. *grimaldii* + *Reteporella* sp. 2 + *Reteporella* sp. 3. However, the bPTP results matched the morphological distinction of species (see below), except for *Reteporella* sp. 4 and *R*. cf. *graeffei*, which are regarded as the same taxon (see Discussion).

Single markers vary in their performance in recovering genetic lineages, although no major conflicts are found in the relationships inferred (Supplementary Material 2: Figs. S1–S3). The mitochondrial COI phylogeny has 23 well-supported nodes in both ML and BI inferences among the *Reteporella* clades, whereas 16S retrieves only five splits supported by ML and BI and two just in the ML reconstruction. Regarding the 28S, BI failed to resolve the tree and ML reconstructions were poorly supported for most splits. Despite the overall high support, COI does not fully resolve the polytomy of the crown group, formed by *R. atlantica, R. oceanica*, and *Reteporella* spp. 5–7. The specimen from Formigas (BRY257) diverges, on average, by 3.4% from *Reteporella* sp. 7 and by 6.3% from *Reteporella* sp. 6 in the COI marker (Supplementary Material 2: Table S2; Fig. S1). In the 16S dataset, the similarity is reversed, with a closer relationship with *Reteporella* sp. 6 (0.9%) than with *Reteporella* sp. 7 (1.2%) (Supplementary Material 2: Table S2; Fig. S2).

### Haplotype network

The TCS network of COI haplotypes (Fig. 3) was used to delineate putative species and evaluate intraspecific haplotype diversity [31], as it was the most informative marker. Overall, haplotypes are distributed linearly and a high proportion of unsampled haplotypes occur in the dataset. In accordance with the terminal branches of the phylogenetic reconstruction, each cluster is considered to be a putative species, such that the analysis distinguishes five formal species (*R. atlantica*, *R. tristis, R. oceanica, R. beaniana*, *R.* cf. *grimaldii*) and seven species that cannot be attributed to any known species (Fig. 2, Supplementary Material 2: Figs S1–3).

In general, a geographical pattern is observed in each cluster, with most haplotypes restricted to a single sample site (private haplotypes). Exceptions are found in two *R. atlantica* haplotypes, shared by Faial and Princess Alice Bank as well as by Terceira and Condor Bank, respectively. Furthermore, one haplotype in *Reteporella* sp. 7 is present in both Santa Maria and São Miguel. The genetic diversity within *R. atlantica* is depicted in a broad network, with eastern localities (Santa Maria and São Miguel/ Sabrina) at one end and samples from Faial at the other. The haplotypes represented by BRY16 (*R. atlantica*, Flores) and BRY257 (*Reteporella* sp. 7, Formigas; see Identification of Morphospecies section below) form isolated clusters, denoting their differentiation from putative conspecifics. Finally, the Mediterranean samples are differentiated in four distinct clusters, according to their geographical origin and assignment to putative species (*R.* cf. *grimaldii* and *Reteporella* spp. 2–4).

### Morphological investigation of ***Reteporella*** species

The identification of the newly collected and sequenced *Reteporella* specimens is based on an analysis of historical type and comparative material collected in the Azores during the late 19^th^ century (e.g. [13, 15, 34]), as well as during the second half of the 20^th^ century (e.g. [16]). A full revision of the established species (*R. atlantica*, *R. gracilis*, *R. oceanica*, *R. rara*, and *R. tristis*) is beyond the scope of the present paper and will be given elsewhere. A summary of the locations where each species is present and distinguishing morphological characteristics of Azorean *Reteporella* taxa is provided in Table 1.

**Table 1:** – Localities and main morphological traits of the *Reteporella* species occurring in the Azores Archipelago

Species	Report of presence	Morphological characteristics
*R. tristis* (Jullien, 1903)	Princess Alice Bank São Jorge Terceira São Miguel Santa Maria	♣ Oval suboral avicularia. ♣ Oval avicularia on the abfrontal side and rarely on the frontal side. ♣ Large spatulate avicularia in some fenestrae.
*R. oceanica* (Jullien, 1903)	Condor Bank Pico	♣ Oval suboral avicularia ♣ Large triangular avicularia on the frontal and abfrontal sides, with three small, subrounded foramina in the extensively calcified palate. ♣ Occasional small avicularia on the abfrontal side.
*R. atlantica* (Busk, 1884) [= *R. gracilis* (Jullien, 1903), likely its junior synonym)]	Flores Condor Bank Princess Alice Bank Chino Bank Faial Pico São Jorge Terceira São Miguel Santa Maria	♣ Three morphotypes of suboral avicularia: small oval, intermediate size triangular, and occasionally a gigantic one in ovicellate zooids. ♣ Oval and elongate triangular avicularia on both the frontal and abfrontal sides.
*Reteporella rara* (Jullien, 1903)	?	♣ Large oval suboral avicularia. ♣ Reticulate surface structure.
*Reteporella* sp. 1	Terceira	♣ Oval suboral avicularium pointing proximomedially. ♣ Triangular avicularia on the frontal and abfrontal surfaces. ♣ Very short pseudosinus next to the suboral avicularium. ♣ Open-branched colony, with cylindrical branches.
*Reteporella* sp. 5	São Miguel	♣ Small oval suboral avicularia. ♣ Large triangular avicularium on abfrontal side, positioned on distinctly swollen cystid, with three small subrounded round foramina in the extensively calcified palate.
*Reteporella* sp. 6	Gigante Bank Terceira	♣ Oval suboral avicularia. ♣ Small oval and larger triangular avicularia on the frontal and abfrontal sides. ♣ Absence of giant avicularia.
*Reteporella* sp. 7	Gigante Bank São Miguel Formigas Santa Maria	♣ Small oval and larger triangular suboral avicularia. ♣ In triangular avicularia, the palatal area distal to the crossbar is wider than in *R. atlantica*. ♣ Absence of giant avicularia. ♣ Pseudosinus length and fenestra size greater than in *R. atlantica.*

#### *Reteporella tristis*

**Fig. 4:**
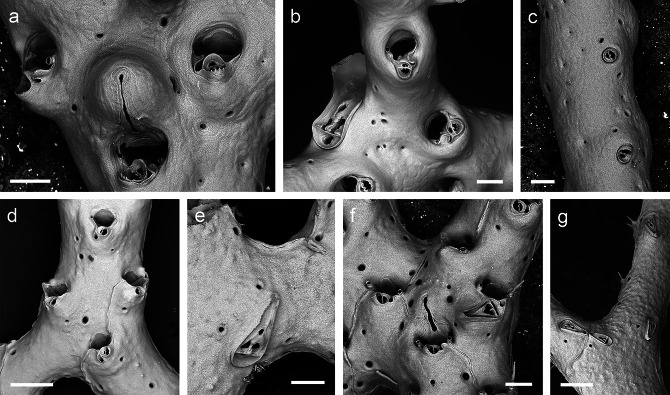
**SEM images of sequenced *Reteporella *species from the Azores. a ***Reteporella tristis*, BRY127, Terceira, 152 m, scale bar 100 μm; **b ***Reteporella tristis*, M151-50341, São Miguel, 314 m, scale bar 100 μm; **c ***Reteporella tristis*, BRY127, Terceira, 152 m, scale bar 100 μm; **d ***Reteporella oceanica*, DOP9869, Condor Bank, 455 m, scale bar 200 μm; **e ***Reteporella oceanica*, DOP9869, Condor Bank, 455 m, scale bar 100 μm; **f ***Reteporella atlantica*, MB6, Santa Maria, 25 m, scale bar 100 μm; **g***Reteporella atlantica*, BRY139, Terceira, 152 m, scale bar 200 μm

*Reteporella tristis* was described from eastern Pico and is characterised by exclusively forming oval avicularia in the suboral position, as well as on the frontal surface (though very rarely) and on the abfrontal side of the colony (Fig. 4A-C). The most conspicuous character, however, is a large spatulate avicularium that is positioned in the proximal axis of some of the fenestrae (Fig. 4B). Sequenced specimens from Santa Maria and Terceira, which form one of the two *Reteporella* clades that are sister to the remaining Atlantic, Mediterranean and Azorean ones (Fig. 2), are attributed to this species, as are additional colonies from São Miguel and Princess Alice Bank that were examined using SEM. All of the specimens, which were recovered from depths between 150 and 1,300 m, are morphologically very similar.

#### *Reteporella oceanica*

The identity and morphology of *Reteporella oceanica*, which was originally described for specimens from both the Azores (around Pico) and the Bay of Biscay, is unclear at present. Its type material could not be studied as syntypes are not present at the MNHN, while those available at the Musée Oceanographique de Monaco are not permitted to be taken on loan. Topotypic material from Pico subsequently identified as *R. oceanica* by Calvet [15] was analysed instead. The species only forms small oval suboral avicularia (Fig. 4D), while this avicularium type is also occasionally present on the abfrontal colony surface, albeit of slightly larger size. Specifically characteristic is a triangular frontal and abfrontal avicularium that is distinctly larger than in the other species described below, and in which the palate is extensively calcified so that the opesia distal to the crossbar is reduced to three small, subrounded foramina (Fig. 4E), whereas in all other species it is Y-shaped. This avicularium morphotype is in agreement with two sequenced specimens from Condor Bank that forms a clade together with *Reteporella* sp. 5 in the phylogenetic tree (Fig. 2). Pending a revision of the species and designation of a lectotype, we here tentatively assign the sequenced specimens and the topotypic material to *R. oceanica*, which is recorded from Pico and Condor Bank in depths between 350 and 455 m.

#### *Reteporella atlantica*

*Reteporella atlantica*, originally described from off northern Pico, is characterised by the presence of three different morphotypes of suboral avicularia: a small oval one, a triangular one of intermediate size (Fig. 4F), and occasionally a giant avicularium with its cystid covering the entire frontal shield of the zooid. While the two smaller types are most frequently observed, the zooids of large parts of a colony may form only one of the types. The giant avicularium apparently occurs only in colony regions with ovicellate zooids, but not all ovicellate zooids form the giant avicularium, and it is also not present in all fertile colony regions or even in entire colonies, such as the ones sequenced herein. Small oval as well as larger elongate triangular avicularia may additionally be budded on the zooid’s frontal surface, particularly during later ontogeny. These two types also occur on the abfrontal side of the colony, while the size of the triangular avicularia is generally slightly larger there (Fig. 4G). *Reteporella gracilis* from off eastern Pico is morphologically very similar to, and cannot be distinguished from, *R. atlantica* at present. The species are likely synonymous.

Most of the analysed specimens of the crown group of Azorean *Reteporella* belong to this morphospecies (Figs. 2 and 3), and the species was found in all three groups of islands and on several seamounts, ranging in depths between 11 m (in Santa Maria) and 820 m (São Jorge Channel, downslope transport cannot be discarded). The single specimen from Flores (BRY16), which shows a higher genetic differentiation, does not display any apparent morphological deviations from the remaining colonies.

#### *Reteporella rara*

*Reteporella rara* is easily distinguished from the other above-mentioned Azorean species by its reticulate surface structure and a significantly larger oval suboral avicularium, among several other differences. None of the recently sampled and sequenced specimens exhibit these features.

Thus, four of the Azorean lineages in the concatenated tree are morphotypes that are distinct from the established species and are regarded as putative new species (*Reteporella* spp. 1, 5, 6 and 7).

#### *Reteporella* sp. 1

**Fig. 5:**
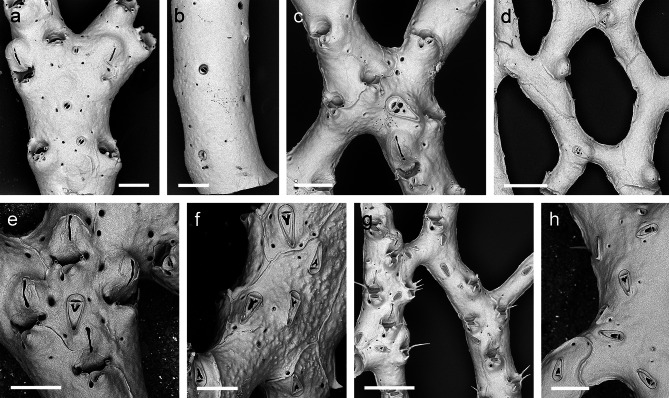
**SEM images of sequenced *Reteporella *species from the Azores. a-b ***Reteporella* sp. 1, BRY116, Terceira, 277 m, scale bars 200 μm; **c ***Reteporella* sp. 5, M151-50333, São Miguel, 311 m scale bar: 200 μm; **d ***Reteporella* sp. 5, M151-50333, São Miguel, 311 m scale bar: 500 μm; **e-f ***Reteporella* sp. 6, BRY146, Terceira, 152 m, scale bars 200 μm; **g ***Reteporella* sp. 7, M151-50332, São Miguel, 310 m, scale bar 500 μm; **h ***Reteporella* sp. 7, M151-50332, São Miguel, 310 m, scale bar 200 μm

The single sequenced specimen from Terceira that is sister clade to *R. tristis* (Fig. 2) produces an open-branched colony, i.e. it is not reteporiform as in other *Reteporella* species (Fig. 5A-B). Its branches are also rather cylindrical while in most *Reteporella* colonies the abfrontal kenozooidal side is somewhat flattened, and the oval suboral avicularium is pointing proximomedially, not proximolaterally as in all others. In contrast to *R. tristis*, the Terceira specimen also forms triangular avicularia on its frontal and abfrontal surfaces. One character in common with that species, however, is the very short pseudosinus next to the suboral avicularium. The open-branched mode of growth observed in the specimen is unique among other Azorean and European *Reteporella* species. Increased sampling effort in the Azores, and particularly in the type locality, is needed to obtain more individuals for a more complete morphological characterization and evaluation of the geographical and bathymetrical distribution of the new taxon, which was recovered from 277 m depth.

#### *Reteporell*a sp. 5

Specimens of *Reteporella* sp. 5 (Fig. 5C, D), which form a clade together with *R. oceanica* (Fig. 2), were only found off São Miguel at 310 m depth. The colonies are characterised by producing swollen cystids on the abfrontal side on which a large triangular avicularium is obliquely placed (Fig. 5D). Frontal and abfrontal avicularium morphology is similar to the one in *R. oceanica*, i.e. with three small, subrounded foramina in the palate, while being slightly smaller in general and the suboral avicularia are also exclusively of the small oval type (Fig. 5C).

#### *Reteporella* sp. 6

The clade comprises populations from Terceira and Gigante Bank, sampled from 150 to 450 m depth, respectively (Figs. 2 and 3). The colonies almost exclusively form oval suboral avicularia as well as small oval and larger triangular avicularia on the frontal and abfrontal surfaces (Fig. 5E, F). However, between the populations there are some differences in, among others, the morphology of the triangular avicularia (they are smaller and the opesia distal to the crossbar is wider and rounder in Gigante Bank specimen, whereas it’s narrower and V-shaped in the colonies from Terceira), and in the position of the ovicell relative to the peristome (the labellum is positioned well above the proximal peristomial margin in the Gigante Bank colonies whereas in the Terceira specimens the labellum is longer and ends below the level of the peristome). The COI divergence levels of 1.5–2% between the two populations thus seem to be reflected in their morphologies.

On the other hand, *Reteporella* sp. 6 is distinguished from *R. atlantica* only by the lack of giant avicularia (as far as we can say from the available material). As entire colonies of *R. atlantica*, or at least large parts thereof, are also missing this type of avicularium, the two species may, despite the considerable divergence of 6.2–10.1% in the COI marker, be morphologically indistinguishable if only non-fertile colonies are available for study.

#### *Reteporella* sp. 7

The specimens are morphologically very close to *R. atlantica*. Both oval and triangular suboral avicularia are, however, generally smaller in this group, while pseudosinus length and fenestra size are distinctly greater than in *R. atlantica* (Fig. 5G-H). Giant avicularia are apparently absent in *Reteporella* sp. 7, and the triangular frontal and abfrontal avicularia are further characterised by a wider uncalcified palatal area distal to the crossbar, whereas in *R. atlantica* its shape is distinctly trifoliate. Most of the analysed specimens were found off São Miguel (310 m) and Santa Maria (150 m), while a single specimen is from Gigante Bank (495 m). Paralleling the molecular results, specimen BRY257 from Formigas (150 m) stands out in having a pseudosinus that is approximately only half the length than in the other colonies, and also in having distinctly smaller fenestrae. In contrast, the triangular frontal and abfrontal avicularia are larger.

### Non-Azorean species

**Fig. 6:**
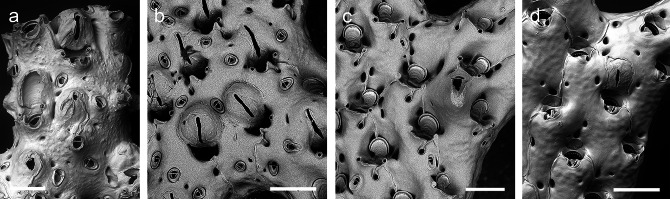
**SEM images of sequenced *Reteporella *species from the NE Atlantic and Mediterranean Sea. a ***Reteporella beaniana*, NHMUK 2022.5.17.1, SSW Iceland, scale bar 200 μm; **b ***Reteporella* sp. 2, DBUA-BRY 68, Gulf of Galéria, Corsica, 80 m, scale bar 200 μm; **c ***Reteporella* sp. 3, DBUA-BRY 64, Pula, Croatia, 5.2 m, scale bar 200 μm; **d ***Reteporella* sp. 4, AW006, Marseille (France), 10–12 m, scale bar 200 μm

The specimen from Iceland is identified as *Reteporella beaniana* (King, 1846) based on comparisons with the description and images provided by Hayward & Ryland [9] (Fig. 6A).

Sister clade of *R. beaniana* is a Mediterranean group of three species that are characterised by triangular suboral avicularia and the occasional presence of a giant suboral avicularium similar to *R. atlantica* whereas small, oval, suboral avicularia are absent. This morphotype has previously been assigned to *R. grimaldii* (e.g. [35]), which was originally described from the Bay of Biscay. The specimen from Spain was sequenced by Orr et al. [7] as *R*. cf. *grimaldii* and we have here adopted that assignment. SEM images of the specimen (BLEED 1851), and all others sequenced by Orr et al. [7] that are included in our analysis, are available from Zenodo (https://zenodo.org/record/5721078#.YZz39VMo_fY). It is further characterised by the presence of small triangular frontal avicularia. The specimen from Corsica (*Reteporella* sp. 2) is distinguished by having rounded frontal avicularia that are usually slightly narrowing distally (Fig. 6B). Differences between this species and the Croatian colonies (*Reteporella* sp. 3, Fig. 6C) are less obvious: for instance, in the latter the small frontal avicularia are usually widening distally and the ovicells produce a larger labellum. All Mediterranean specimens are, however, very likely specifically distinct from the nominal species. The peculiar thickening at the base of the ovicell, which is well visible in the holotype of *R. grimaldii* (see [36]: fig. 5D, E), is missing in the Mediterranean colonies or at least not visible in frontal view.

The voucher specimen from Marseille (*Reteporella* sp. 4) lacks any frontal avicularia, and has, besides oval suboral avicularia and relatively large areolar pores of variable shape, no distinct features (Fig. 6D). It cannot be attributed to any known species at present. It is genetically almost identical with, and morphologically very similar to, the specimen sequenced and identified as *Reteporella* cf. *graeffei* by Orr et al. [7] (BLEED 1059), which is, however, supposedly from Australia. *Reteporella tuberosa* from Australia (BLEED 420B) is genetically significantly different from other species of the genus and in need of revision.

## Discussion

Oceanic islands that originate *de novo* from volcanic eruptions, which allow for a precise estimate of their origin via radiometric dating, are ideal places to study patterns and processes of evolution ever since Darwin. Depending on their distance to other islands or the continental shelf, colonisation of oceanic islands may prove difficult, not just for terrestrial but also for marine organisms, particularly those with short-lived larvae [37]. Among the many dispersal strategies of organisms with non-planktotrophic larvae (for a review see [38]), which include all brooding bryozoans, rafting is the most relevant for epibenthic shallow-water taxa in temperate Atlantic regions [39–41]. Adding to this difficult quest, the Azores Archipelago sits in a particular geographic and oceanographic setting in the middle of the North Atlantic, with prevailing eastwards-flowing sea-surface currents (see [29] for details). It is remarkable that, despite the Gulf Stream and similarly to other marine and terrestrial taxa, non-planktotrophic Azorean bryozoans have their closest relatives in the NE Atlantic or the Mediterranean Sea, providing another example of the so-called “Azorean Biogeographical Paradox” [23–25]. Dispersal limitations imposed by distance and short-lived larvae would be expected to cause a low bryodiversity in the Azores, although if isolated long enough insular populations may evolve into new species. The Azores Archipelago, with its numerous islands and varying distances to each other, may be regarded as being particularly prone to adaptive radiations. One would, therefore, expect that the islands had originally been colonized by a few shallow-water taxa, more prone to chance events of dispersal, which have then diversified and spread into deeper waters, producing a larger number of endemic species. Its proximity to the mid-Atlantic ridge as well as to older seamounts in the region (e.g. the Meteor-Atlantis seamount complex in the south), may offer additional source regions for bathyal taxa.

Therefore, by extending the previously limited sampling of bryozoans in the Azores, and by the application of genetic data coupled with state-of-the-art taxonomic criteria and imaging methodologies, more species than hitherto recognised might be revealed, including unknown native and endemic taxa [23].

### Marker performance and concordance with morphospecies

To examine the diversity of the genus *Reteporella* in the Azores we performed a multi-marker phylogenetic reconstruction complemented with a morphological characterization of the putative species defined. The mitochondrial COI is the marker performing best in the phylogenetic reconstructions and contributes to most of the phylogenetic signal of *Reteporella* species. The region of the 28S marker analysed appears to have a low differentiation power of *Reteporella* diversity, and has previously been pointed out as poorly informative [42, 43]. The genetic groups identified with 28S are consistent with the major mitochondrial lineages and morphospecies of *Reteporella*, with diagnostic point mutations across its 334 bp. The poor performance of this marker has been noted in other genera, such that another region of 28S or a different nuclear marker is required to increase the phylogenetic signal and resolve the nuclear phylogeny of *Reteporella* [43–45].

Except for *Reteporella* sp. 6, which is morphologically extremely similar to *R. atlantica* and may be regarded as a cryptic species, species boundaries recovered with bPTP match the morphospecies defined in this study, whereas mPTP underestimates the number of lineages by clustering taxa that have been distinguished by morphological characters and mitochondrial divergence (e.g. *R.* cf. *grimaldii* from Spain, *Reteporella* sp. 2 from Corsica, and *Reteporella* sp. 3 from Croatia). Incongruent performance of these tree-based species delimitation methods has been previously linked to higher substitution rates, unevenness of sampling, different population sizes among species, ongoing gene flow, or unresolved nodes, leading to over-splitting or over-lumping of the taxa [46].

In understudied organisms, such as bryozoans and many other marine phyla, DNA barcoding is an important tool to reveal hidden biodiversity, particularly cryptic species and sister taxa with overlapping geographic ranges. It can also be used to test the extent of phenotypic variability among individuals genetically assigned to the same taxon and the species status [47], as is the case of *R. atlantica* in this study. Ultimately, it reveals overlooked taxa in repositories, such as in Macaronesian marine macroinvertebrates [48, 49], indicating the necessity for taxonomic revisions. In these poorly known groups, however, the barcoding task can be hampered by unknown levels of DNA sequence divergence, which vary greatly among taxa. Defining this “barcode gap” is challenging and implies a priori knowledge of morphology and phylogenetic relationships of the studied group, otherwise it can lead to over- or underestimation of the real diversity present in the dataset [43, 50–52].

Of the markers analysed, the mitochondrial COI seems to be the best at delimiting genetic lineages and putative species. In the haplotype networks observed in our dataset, the separation of clusters at the 95% connection limit has been suggested by some to indicate deep differences and putative new species (e.g. [31]). Such interpretations have to be applied cautiously, as the overall molecular divergence levels in bryozoans are high and often do not obey the traditional clustering threshold of 97% identity for species [47, 52, 53]. Among *Reteporella*, the overall concordance between molecular analyses, species delimitation analyses with the bPTP algorithm, and morphospecies inferred in this study suggests the potential adequacy of the 3% COI cut-off in this group [50, 54, 55].

In recent years a wave of criticism has grown regarding the improper use of DNA barcoding approaches alone to formally recognize species without further evidence from morphology or ecology (see [45, 56, 57]). Therefore, an integrative approach that encompasses molecular, morphological, and even ecological data is regarded as the future of taxonomical descriptions [44, 57]. This is especially important for cheilostome bryozoans, considering that this speciose order is the result of a rapid radiation starting in the mid-Cretaceous [58], which can obscure the phylogenetic signal of extant divergences [44]. Due to the high diversity of phenotypes and genotypes, relying on molecular analysis as a backbone to identify genetic lineages for subsequent morphological comparisons and characterization has been shown to be a fruitful approach in cheilostome bryozoans [43, 51, 52, 59–61]. In this study, we report the mutual support between morphospecies and genetic species in seven lineages and four established *Reteporella* species, following findings in other cheilostomes, namely *Stylopoma, Rhynchozoon, Celleporella*, among others [51, 52, 59, 62, 63]. On the other hand, within certain *Reteporella* species, some well-supported branches do show morphological character differences that distinguish populations, while other genetically distinct populations are morphologically identical (see below). Therefore, we reinforce that molecular approaches have the potential to reveal unknown diversity, although morphological characterization and taxonomic validation by experts is mandatory. The idea that new morphospecies tend to be overlooked by classical taxonomy is often not true in understudied organisms, but rather a consequence of the low number of taxonomists specialized in these groups. Nowadays it certainly seems that molecular analyses provide faster and cheaper results to uncover biodiversity, but it is the unbalance between the number of molecular biologists and classical taxonomists that hamper the complete description of diversity in these poorly studied groups [64].

### Phylogeny of the Phidoloporidae

Despite the low resolution of 16S and 28S markers, no major conflicts are found between the gene trees, each marker contributing to the overall topology of the concatenated tree. Inconsistent placement of taxa and low support of node split among the gene trees are expected, as the markers are likely to have distinct evolutionary rates [44]. The low accuracy of the reconstruction can also be related to the outgroup choice (*Celleporaria* cf. *fusca*), which is often problematic as it is limited to the existing phylogenetic frameworks and sequence data available in GenBank. Nevertheless, the inclusion of phylogenetically closer phidoloporids did not further improve the support of the phylogenies. Albeit a detailed interpretation of the relationships with other phidoloporid taxa is out of the scope of this study and will be given elsewhere, we remark that the clades inferred here are in accordance with Orr et al. [7].

The addition of non-*Reteporella* phidoloporid taxa to the dataset allowed the estimation of high intergeneric genetic distances within the family (17–25% in COI). Similar divergence levels were inferred between members of the genus *Reteporella*. This is especially true for the Atlantic and Mediterranean *Reteporella* lineages [(*Reteporella* sp. 1 + *R. tristis*), (*R. beaniana* + *R*. cf. *grimaldii* + *Reteporella* sp. 2 + *Reteporella* sp. 3), (*R. atlantica*, *Reteporella* spp. 5–7)], with divergence levels ranging from 16 to 20% that suggest a considerable independent history. Such high levels of divergence hint either at the need to split *Reteporella* clades into several new genera, as generically distinct yet morphologically similar taxa seem to be currently classified under the same rank, or to lump other phidoloporids into the same genus. Phylogenetic reconstructions of the family Phidoloporidae, as well as morphological and morphometric analyses, are needed to support the reclassification and define diagnostic characters.

### The genus ***Reteporella*** in the Azores

#### Diversity

Of the four established Azorean *Reteporella* species, three were studied – *R. atlantica, R. oceanica*, and *R. tristis* – being *R. gracilis* likely a junior synonym of *R. atlantica* and the *R. rara* morphotype not among the sampled specimens. Besides the record of several other nominal species that have their type locality outside the Azores, which need to be revised, this study adds another four putative new species to the archipelago’s bryodiversity, supported by phylogenetic reconstructions, haplotype networks, and distinctive morphological characters. The underestimation of bryozoan diversity in the Azores has been previously pointed out to be a consequence of sporadic sampling, focused mostly on the islands of Faial, Pico and São Miguel, and the lack of taxonomic revisions using modern imaging techniques [23]. The sampling effort for this study was enhanced, with a considerable extension of the geographic and bathymetric ranges, allowing to reduce this bias and revealing new species in the region. Genetic diversity levels higher than reported are frequently estimated in taxonomic revisions that include molecular studies, often corroborating distinctions previously suggested based on morphological differences [43, 48, 51, 63]. Intra-specific morphological variation and genetic divergence are not only associated with genetic drift but also with geographical and ecophenotypic factors [59, 65]. The observation of the full range of morphological variation in *Reteporella* and other erect bryozoans is limited by destructive sampling methods [4, 6, 59]. Thus, in order to record the full range of intraspecific variability and relate it with genetic divergence levels, further morphometric work is needed in the morphospecies of *Reteporella* here distinguished, with an increased number of colonies and colony sections to be scrutinized with SEM.

The predominantly linear haplogroups in the haplotype networks suggest stable, isolated lineages with infrequent gene flow [66]. As in *Celleporella hyalina* in the NE Atlantic [51], most COI haplotypes across *Reteporella* species are classified as private, i.e., they are restricted to a single locality. In most cases, this is either an artefact of low sampling density [e.g., in the seamounts Chino Bank, Gigante Bank, Condor Bank) or a result the inclusion of rare species (e.g., *R. oceanica*), but even in the widely distributed *R. atlantica* (Busk, 1884) most haplotypes are private. The high incidence of unsampled haplotypes in the Azorean *Reteporella* lineages from this study indicates high diversity richness that has not yet been sampled or analysed [32], whereas the robustness of poorly supported nodes in the phylogenetic reconstruction can probably be increased by including crucial unsampled taxa [52]. Both observations highlight the importance of a broader sampling geographically and particularly bathymetrically.

Overall, there is a marked phylogeographical pattern in each Azorean *Reteporella* clade, congruent with the low dispersal abilities of a non-planktotrophic developer [51, 66, 67]. Owing to the short duration the larvae of *Reteporella* spend in the water column before settlement, strong philopatry and isolation by distance patterns are common [5, 67–70], particularly in an archipelago such as the Azores in which the habitable islands (hard substrata) are separated by uninhabitable deep-sea stretches (sandy to muddy sediments). A pattern of isolation by distance (i.e., increase of differentiation with geographical distance between the islands) is noticeable in the species sampled (*R. atlantica, R. tristis, Reteporella* spp. 6 and 7).

As aforementioned, all the species proposed in this study are supported by morphological and molecular data but some relationships are noteworthy. The phylogenetic position of the clade formed by *R. oceanica* and *Reteporella* sp. 5 is not resolved in any of the phylogenies, suggesting a missing link in the dataset. Still, considering the distinct morphological differences between the clades, their geographic separation (central group of island in the former, eastern group in the latter), and results of the species delimitation analyses with the bPTP algorithm, we believe these sister species to be a good example of the 3% barcode gap [50, 54, 55], although more samples are necessary to assess the real levels of molecular differentiation between them. *Reteporella* sp. 6 and sp. 7 display high mitochondrial divergence levels (5.7–6.3%), which have been linked to reproductive incompatibility [47, 63], and should thus be considered different species. One sample of *Reteporella* sp. 7 from Formigas (BRY257) displays morphological distinctions in most of the parameters evaluated (see Results section “Identification of morphospecies”) and is inconsistently assigned to different species depending on the markers analysed. Erroneous placement of this sample within *Reteporella* sp. 7 cannot be discarded, although the unavailability of more samples from Formigas hampers identity testing. Despite a significant genetic distance, *Reteporella* sp. 6 differs from *R. atlantica* only in the absence of giant frontal avicularia. Colonies or colony fragments of *R. atlantica* without these avicularia are thus indistinguishable from *Reteporella* sp. 6, which may therefore be regarded as a cryptic species. Future work and a richer dataset of specimens from *Reteporella* sp. 6 and sp. 7 are needed for a detailed characterization based on molecular and morphological characters.

*R. atlantica* is the most abundant species included in the dataset and it is likely found in the Azores Archipelago between 11 and 820 m in all island groups. Despite extensive scuba diving efforts in islands other than Santa Maria, shallow-water *Reteporella* colonies were not found, being the next shallowest record of *R. atlantica* reported at 60 m depth off Faial by Wisshak et al. [71]. Notwithstanding the absence of morphological distinctions, one specimen from off Flores (BRY16) shows, to some extent, mitochondrial differentiation to the remaining *R. atlantica.* The veracity of these genetic divergence levels was verified by two independent rounds of sequencing for both mitochondrial markers, all resulting in good quality sequences, confirming the genetic distinctiveness of this specimen. Ecological surveys to characterize the habitat at 150 m around Flores and to obtain more samples similar to BRY16 are necessary to clarify whether this constitutes a distinct lineage of *R. atlantica* or if it is just a product of natural genetic drift in the population [65]. Population genetic studies are needed to clarify the diversity and distribution of *R. atlantica* in the Azores.

#### Biogeography and bathymetric distribution

The Azorean taxa are divided into two clades: *R. tristis* and *Reteporella* sp. 1 originate from an earlier diverging ancestor of unclear geographic origin, whereas *R. atlantica, R. oceanica*, and *Reteporella* spp. 5–7 have split more recently. The latter, closely related to the North Atlantic *R. beaniana* and the Mediterranean *R.* cf. *grimaldii* and *Reteporella* spp. 2–4, constitutes the crown clade and hints at the occurrence of a radiation in the archipelago. The moderate support of the clade might be due to the existence of other related species in the archipelago, which have not been sampled or that went extinct over time. The existence of two Azorean clades argues for at least two colonization events of *Reteporella* taxa in the remote Azores Archipelago. The close relationship between Mediterranean and Azorean taxa constitutes yet another example of the so-called “Azorean Biogeographical Paradox” [24, 25], but inferences cannot be made regarding the direction and timing of past colonization events. The inclusion of more of the c. 20 known species from the Mediterranean Sea and North Atlantic in future phylogenies of the genus or the family Phidoloporidae [3, 72] will allow a better understanding of this complex evolutionary history. Moreover, the lack of samples from the western North Atlantic has certainly affected the results of our analysis, although the genus is rare and species-poor in this region.

Brooding bryozoans such as *Reteporella* are unlikely to reach remote islands during their short planktonic larval stage, thus rafting of shallow water populations on floating algae and other suitable substrata has to be regarded as the most plausible means of transport [38, 39, 73, 74]. One would therefore expect the shallow-water populations/species to be ancestral but, except for *R. atlantica*, *Reteporella* species in the Azores mostly inhabit ecosystems deeper than the shallow euphotic zone where the potential rafts (algae and seagrass) occur. For this reason, source populations from older seamounts near the Azores or the mid-Atlantic ridge may need to be taken into consideration.

Considering the geographical and oceanographical setting of the Azores in the central North Atlantic, isolated by over 1,000 km from the nearest land, we believe that most (if not all) *Reteporella* species are endemic to the archipelago. It is very likely to also apply to the taxa historically referred to nominal species with type localities outside the archipelago (e.g. *R. mediterranea* by Calvet [15] or *R*. cf. *sparteli* by d’Hondt [16]). Single island marine endemic species (SIMEs) are, however, rare in oceanic islands [27, 41]. Although our dataset suggests *Reteporella* sp. 1 (Terceira) and *Reteporella* sp. 5 (São Miguel) as potential SIMEs, this might be due to sampling bias and further work is necessary to clarify their status. The high degree of endemism of Azorean bryozoans at species-level (see also [21, 75, 76]), and even at generic-level (seven genera, including the newly proposed genus derived from *Reteporella* sp. 1 and *R. tristis* ), contributes to the biogeographic uniqueness of the Azores ecoregion [27].

In some localities across the Azores Archipelago (Terceira, Santa Maria, São Miguel), different taxa co-occur in sympatry, especially below 150 m depth. Terceira was the locality with the highest number of species identified (four): *R. atlantica, R. tristis, Reteporella* spp. 1 and 6 in depths between 150 and 280 m. The geographic distribution of *Reteporella* sp. 7 suggests that the species may also be present in the island but remained unsampled. Located in the Central Group, this island is in a privileged location to be colonized and receive larvae from both the western and eastern localities [77]. Off Santa Maria and São Miguel islands, a number of *Reteporella* species comparable to Terceira also occur in sympatry (*R. atlantica, R. tristis, Reteporella* sp. 7 in both islands, plus *Reteporella* sp. 5 in São Miguel). Being the easternmost islands of the archipelago, it is not surprising that high diversity is encountered here, as under the eastbound sea-surface circulation, bryozoan larvae/rafts are likely to divert to this area of the archipelago [66, 77–79].

In Flores only one species (*R. atlantica*) was present, but this low diversity can be an artefact of the sampling at only one site and depth (150 m). More species are likely to occur at other bathymetries, although an overall lower diversity would not be surprising considering the westernmost position of this island in the archipelago, which, under the eastbound circulation regime, does not favour retention of particles, and thus larvae, in the area [66, 77, 79].

The greatly varying distances between islands, seamounts and groups of islands (e.g. 6 km between Faial and Pico within the central group; 220 km between Flores in the western group and the nearest island of Faial in the central group) promotes isolation of populations in some of the islands during normal times, i.e. under the prevailing east to southeast flowing Azores Current. *Reteporella* populations in distant islands that exceed the range that larvae can travel in the water column prior to successful settlement and metamorphosis, may thus be subject to prolonged evolution in isolation. Rare chance events such as strong storms or hurricanes [80, 81], or the formation of eddies [77, 79], may enhance sea-surface current speed or even induce a change in current direction, and thus connect separated populations or bring together distinct species [29].

In addition, during glacial periods the distance between islands and seamounts (the shallower ones of which turning into islands, e.g., Princess Alice Bank) was slightly reduced owing to the sea-level low stand, theoretically favouring an exchange of populations between islands then [82]. Changes in the latitudinal position, strength and direction of the Azores Current between glacial and interglacial periods may have contributed to the complex genetic and geographic pattern observed in some of the species today [41, 83].

The Azorean seamounts (Chino Bank, Condor Bank, Gigante Bank, and Princess Alice Bank) also seem to yield a considerable diversity of *Reteporella*. The deep waters separating the seamounts and islands have traditionally been regarded to constitute vast, stable, and homogeneous habitats without barriers to dispersal [84]. Today, it is known that even deep-water ecosystems are influenced and partitioned by currents and physico-chemical characteristics of the water masses, whereas seamounts are complex geomorphological structures under multi-scale dynamics, related to circulation and upwelling-downwelling patterns [85, 86 and references therein].

Moreover, some of the seamounts in the vicinity of the Azores, such as the Great Meteor Bank to the south, are considerably older than the islands [87], and may have been islands themselves during the late Miocene when the oldest Azorean island, Santa Maria, originated. It is unknown when *Reteporella* first occurred in the Azores. The early Pliocene colonies from Santa Maria reported as *Reteporella* sp. [88] turned out to belong to the genus *Schizoretepora* upon closer inspection (BB, pers. observ.).

As all these factors greatly affect biodiversity and biogeographical patterns, a full characterization of the islands’ and seamounts’ assemblages and biogeographical affinities of *Reteporella* and other marine invertebrates would benefit from detailed future studies in the area from a multi-disciplinary perspective.

#### Non-Azorean ***Reteporella*** species

In addition to *R. tuberosa* and *R. beaniana*, three putative new species of *Reteporella* from the NE Atlantic and the Mediterranean are detected in this study, while *R.* cf. *graeffei and R.* cf. *grimaldii* are presumably also specifically distinct from their nominal species. The well-supported phylogenetic position of *R. tuberosa* in the reconstructions, clustering with *Triphyllozoon* as in the analyses of Orr et al. [7], calls for the need to revise its generic classification. Two clades of Atlantic and Mediterranean *Reteporella* arise in the reconstructions: (1) *Reteporella* sp. 4 and *R.* cf. *graeffei*, which are regarded as the same taxon and are closely related to the Azorean crown group, and (2) *R. beaniana, R.* cf. *grimaldii*, and *Reteporella* spp. 2 and 3.

The presence of the same species in Mediterranean waters (*Reteporella* sp. 4) and the tropical waters of Camden Sound (Western Australia; *R.* cf. *graeffei*) is surprising and requires further investigation. *Reteporella graeffei* is not well defined, as its types have never been revised, and the SEM images provided by Orr et al. [7] show that the sequenced specimen differs significantly from the morphotype that is usually regarded as *R. graeffei* (cf. [89, 90]). Although the colony imaged by Orr et al. [7] has two types of frontal avicularia, its overall morphology, including the presence of large areolar pores, agrees with *Reteporella* sp. 4 from Marseille. The absence of frontal avicularia in the small fragment available of *Reteporella* sp. 4 may, as in *R. atlantica*, may be due to intracolonial variability. This taxon, represented by *Reteporella* sp. 4 and *R.* cf. *graeffei*, is thus either non-indigenous or one of the specimens may have been mislabelled.

Within the second clade, differences between the Mediterranean *R.* cf. *grimaldii* and *Reteporella* spp. 2 and 3 are not observed in the nuclear marker. We tentatively suggest that *Reteporella* spp. 2 and 3 are distinct species due to the divergence levels in COI and the presence of morphological character differences that may indicate potential reproductive isolation. *Reteporella* spp. 2 and 3 were sampled on opposite sides of the Italian Peninsula, respectively in the Adriatic and Ligurian Seas. These localities are separated not only by emerged land but also by complex sea-surface circulation patterns and oceanographic fronts (Sicily Channel and Otranto Strait), which are known to increase genetic differentiation in marine invertebrates with short-lived larvae and reduced adult mobility [91]. Nonetheless, the decision of whether these lineages constitute different morphospecies requires additional SEM analysis and sequencing of colonies from geographically intermediate localities (i.e., southern Italy). The recognition of putative new Mediterranean *Reteporella* species highlights the need to revise them, as more species than hitherto acknowledged are present in the region.

## Conclusion

This study constitutes the first detailed genetic analysis of the genus *Reteporella*, contributing to the catalogue of genetic data of this often-overlooked phylum. Over 100 samples collected in a wide geographical and bathymetrical range in the Azores revealed a higher diversity than previously thought, with the identification of four putative new *Reteporella* species in the Azores and three in the Mediterranean. An overall concordance between the morphospecies and COI data, with a threshold of c. 3%, is demonstrated for *Reteporella*. To prove the status of *Reteporella* morphospecies as good biological species would require mating experiments [47, 59], which is, however, not an easy task in this genus.

The samples included in this study already grant considerable geographical coverage in the Azores, but further sampling in more regions and over greater depth ranges is necessary to uncover the real diversity of the genus *Reteporella* in the Azores. Of high importance is the study of seamounts, with the potential to yield unique biodiversity and areas of sympatric occurrence, as observed in Gigante Bank. The clarification of biogeographical questions would benefit from the application of genome-wide markers such as microsatellites to evaluate connectivity and differentiation at different temporal and spatial scales. An integrative approach with several lines of evidence – morphometrics, molecular inferences drawn from different markers, life-history traits, and ecology – is the future of taxonomy and species delimitation, and should be followed in future studies to properly characterise the diversity of *Reteporella*.

## Methods

Here we present phylogenetic reconstructions of *Reteporella* species sampled across different benthic habitats in the Azores and adjunct seamounts, complemented with samples from the Mediterranean Sea and North Atlantic (Fig. 1). Phylogenies were based on partial sequences of genes cytochrome *c* oxidase subunit I (COI), small subunit mitochondrial ribosomal RNA (16S) and large subunit nuclear ribosomal RNA (28S). Molecular clades are interpreted in combination with morphological data to characterize the diversity and biogeographic distribution of *Reteporella* species in the Azores Archipelago.

### Sampling and morphological characterization

*Reteporella* specimens from several locations around the Azores Archipelago were either recently sampled by the authors or sub-sampled from collection material (Fig. 1, cf. Supplementary Material 1: Table S1 for further details). During the scientific cruise M150 BIODIAZ “Controls in benthic and pelagic BIODIversity of the AZores” with the German RV *Meteor* in 2018, *Reteporella* specimens were collected around the islands of Flores, Terceira, and Santa Maria, from depths of 140–280 m with a box corer as well as Henning- and Shipek-Grabs [92]. Shallow-water sampling around the island of Santa Maria was carried out by SCUBA diving by LB and BB at depths between 10 and 30 m in 2015 and 2019. All collected samples were preserved in ethanol > 96% and stored at 4ºC or below.

Voucher specimens of each Azorean lineage were dried and bleached overnight in 10% diluted household bleach to remove soft tissues for SEM inspection. This took place at the University of Vienna (Austria) with a JEOL JCM-6000Plus Benchtop, at the Natural History Museum London (NHMUK) with a LEO 1455VP and a JEOL IT500, at Senckenberg am Meer in Wilhelmshaven, Germany (SaM) and the Muséum National d’Histoire Naturelle in Paris, France (MNHN) with a Tescan VEGA SEM, and at CIBIO-Açores, Portugal with a Phenom Pro X. Dried reference material is kept at the institutes mentioned above as well as at the Biologiezentrum of the Oberösterreichische Landes-Kultur GmbH in Linz (OLL; collection Invertebrata except Insecta).

The morphology of historical type and topotypic material of Azorean *Reteporella* species was examined at the NHMUK and the MNHN. Putative Atlantic and Mediterranean species and lineages, as defined with molecular approaches and used for comparison, were identified using published literature (e.g. [3, 9, 35]). Species descriptions are not given here in detail but will be provided in future works in which the taxa are revised or newly introduced. The recently described *Reteporella azorensis* Souto, 2019 is not considered here, as its morphology (e.g. ovicell with a proximal fissure) suggests a placement in the genus *Schizoretepora* Gregory, 1893.

### Molecular work

Total genomic DNA (gDNA) was extracted from clean colony fragments with the column-based commercial kit PureLink® Genomic DNA (Invitrogen™), following the manufacturer’s instructions after homogenization of the mineralized skeleton with metallic pestles. DNA samples, in a final elution of 40µL, were analysed using a Nanodrop® 2000 to assess DNA quality and quantity. DNA integrity was checked using agarose gel electrophoresis (0.8% w/v). PCR amplification of COI was performed in 25 µl volumes, using 3 µl of gDNA (some samples required 1:2 dilution), 10 x MgCl_2_ free buffer, 2.5 mM MgCl_2_, 0.2 mM dNTP, 10 µM of each primer, 0.1 µg/µL bovine serum albumin (BSA, Promega) and 0.3 U Platinum Taq DNA polymerase. Cycling conditions were as follows: 94 °C for 5 min; 35 × (94 °C for 30 s, 53 °C for 45 s, 72 °C for 1 min); 72 °C for 10 min. 16S and 28S markers were amplified in 20 µL volumes, using 2 µl of gDNA, 10 µl of QIAGEN Multiplex PCR Master Mix (Qiagen, CA, USA), and 4 µl of each 2 µM primer. Cycling conditions were as follows: 95 °C for 15 min; 35 × (95 °C for 30 s, annealing for 1 min, 72 °C for 30 s); 72 °C for 10 min. Details on the primers and annealing temperatures are presented at Table 2. A reverse COI primer (coiR_ret) was newly designed with Primer3 as implemented in Geneious 8.1.9 [93] and checked for secondary structures with the web-based tool Netprimer (http://www.premierbiosoft.com/netprimer/). PCR products were evaluated using 2% (w/v) agarose gels with GelRed (DNA fluorescent dye, BioTarget™). Purification and bi-directional Sanger sequencing were performed by Genewiz (Azenta), Leipzig, Germany.

**Table 2:** **Primers used for PCR amplification and sequencing of the markers COI, 16S, and 28S.** T_ann_ = annealing temperature

	Name	Sequence		T_ann_		Reference		
COI	cox1F_prifi	TTGRTTYTTTGGWCAYCCHGAAG	53ºC		[60]			
	cox1R_prifi	TCHGARTAHCGNCGNGGTATHCC	53ºC		[60]			
	coiR_ret	GCTAGHCCTAGGAARTGTTGA	53ºC		This study			
16S	16Sar-L	CGCCTGTTTATCAAAAACAT	52–58ºC *		[94]			
	16Sbr-H	CCGGTCTGAACTCAGATCACGT	52–58ºC *		[94]			
28S	28SC1	ACCCGCTGAATTTAAGCAT	55ºC		[95]			
	28SC2	TGAACTCTCTCTTCAAAGTTCTTTTC	55ºC		[96]			

* T_ann_ varied in this range for different species.

All chromatograms were inspected with Geneious v. 8.1.9. COI data were checked for premature stop codons and frameshift mutations using AliView v1.26 [97]. Living in close association with other biota complicates the production of *bona fide* genetic data for the target bryozoan species, inhibiting PCRs and/or causing amplification of non-target contaminant DNA [7, 60]. Thus, contaminant sequences of dubious origin were identified and excluded from our datasets, based on nucleotide alignments and sequence identity searches with BLASTN [98]. All verified *bona fide Reteporella* sequences generated during this study were deposited on GenBank (https://www.ncbi.nlm.nih.gov/genbank/; COI: OP070973 to OP071039, 16S: OP070973 to OP071039; 28S: OP070836 to OP070924). Publicly available mitogenomes and 28S sequences of several phidoloporids – *Reteporella beaniana* (King, 1846), *R.* cf. *grimaldii* (Jullien, 1903), *R.* cf. *graeffei* (Kirchenpauer, 1869), *R. tuberosa* Hayward, 2000, *Iodictyum yaldwyni* Powell, 1967, *Iodictyum violaceum* Hayward, 2004, *Hippellozoon novaezelandiae* (Waters, 1895), *Phidolopora avicularis* (MacGillivray, 1883), and *Triphyllozoon arcuatum* (MacGillivray, 1889) – were added to the dataset; details are available in Supplementary Material 1: Table S1.

### Phylogenetic reconstructions

COI and 16S boundaries of unannotated mitogenome GenBank records were identified using the MITOS WebServer [99] (https://mitos.bioinf.uni-leipzig.de/index.py). Alignments of rRNA genes were constructed using the MAFFT web server [100] with the Q-INS-I model, which considers secondary RNA structure [101], whereas for the protein-coding COI we used the Clustal Omega algorithm from Web Services by EMBL-EBI [102]. MEGA11 [103] was used to reduce alignments to single haplotypes, and to calculate uncorrected (p) distances. The best-fit partitioning schemes and models of molecular evolution were assessed with the software PartitionFinder v1.1.1 [104], following the Akaike Information Criterion [105]. To minimize the saturation effects of codon positions and to account for codon position-specific rates of molecular evolution, COI data were partitioned by codon position [106, 107]. Poorly aligned positions in the rRNA gene alignments were removed with GBlocks v0.91b with default settings, available at http://phylogeny.lirmm.fr/ [108, 109]. The models of molecular evolution for the COI data were set as GTR + I (1st codon position), F81 + I (2nd codon position), and GTR + G (3rd codon position), whereas GTR + I + G was the model chosen for the 16S and 28S markers. We used published data for *Celleporaria* cf. *fusca* (Busk, 1854) as the outgroup, as *Celleporaria* Lamouroux, 1821 was found to be the sister taxon to a derived clade of cheilostomes that included phidoloporids (Waeschenbach, unpublished data).

Phylogenetic analyses were conducted on single-gene partitions and the concatenated dataset (COI + 16S + 28S). Analyses were carried out using Bayesian inference (BI) and maximum likelihood (ML) methodologies. BI analyses were conducted in MrBayes v3.2.7 software [30]. Two independent runs, each with four chains, were completed for 2 × 10^7^ Markov Chain Monte Carlo (MCMC) generations. The heating chains parameter was set to 0.25 and the burn-in was 25%. Convergence between runs was determined once the average standard deviation of split frequencies was below 0.01. Trees and parameters were sampled every 1,000 generations. For the concatenated dataset, only BI analysis was performed. ML analyses were performed in W-IQ-Tree web server [110], set for 1,000 ultrafast bootstraps (UFBoot) [111], SH-aLRT branch tests [112] for 1,000 replicates, and partitioned models [113] for the individual gene partitions. Trees were visualized, rooted, and edited with FigTree v1.4.3 software.

Species delimitation analyses using Bayesian implementation of the Poisson Tree Process model (bPTP; https://species.h-its.org) [114] and a multi-rate PTP (mPTP; https://mptp.h-its.org/#/tree) [115] were carried out on the single locus COI maximum-likelihood tree. The bPTP analysis was carried out over 100,000 MCMC generations with a thinning of 100 and a 25% burn-in, whereas default settings were applied for the mPTP analysis. PTP approaches model speciation in terms of number of substitutions, such that more substitutions are expected in interspecific branching events [114]. The mPTP process is a recent improvement of PTP that incorporates intraspecific divergence caused by different evolutionary histories or sampling biases of the species [115]. To further evaluate the relationships among COI haplotypes and delineate putative species, a statistical parsimony haplotype network at the 95% connection limit was estimated with the software TCS v1.21 [32] and edited with tcsBU web-based program [33].

## Electronic supplementary material

Below is the link to the electronic supplementary material.

Supplementary Material 1

Supplementary Material 2

## Data Availability

The genetic data underlying this article is available in the GenBank Nucleotide Database at https://www.ncbi.nlm.nih.gov/genbank/ and can be accessed with the GenBank accession numbers OP070836 – OP070924 (28S), OP070973 – OP071039 (16S), and OP086099 – OP086207 (COI).

## Abbreviations

16S: Small subunit mitochondrial ribosomal RNA
28S: Large subunit nuclear ribosomal RNA
AZO: Azores
BI: Bayesian inference
bPTP: Bayesian implementation of the Poisson Tree Process
CB: Condor Bank
ChB: Chino Bank
COI: Cytochrome *C* oxidase subunit I
COR: Corsica
CR: Croatia
ES: Spain
FAI: Faial
FLW: Flores
FOR: Formigas
GB: Gigante Bank
GTR: General Time Reversible
ICE: Iceland
KAT: Kattegat
MCMC: Markov Chain Monte Carlo
ML: Maximum Likelihood
MNHN: Muséum National d’Histoire Naturelle
mPTP: Multi-rate Poisson Tree Process
MRS: Marseille
NHMUK: Natural History Museum London
OLL: Biologiezentrum of the Oberösterreichische Landes-Kultur GmbH
PAB: Princess Alice Bank
rRNA: Ribosomal ribonucleic acid
SaM: Senckenberg am Meer in Wilhelmshaven
SEM: Scanning electron microscopy
SIME: Single island marine endemic species
SMA: Santa Maria
SMG: São Miguel/Sabrina
TER: Terceira
UFBoot: Ultrafast bootstraps
