# Supplementary material for: Morphospecies and molecular diversity of ‘lace corals’: the genus Reteporella (Bryozoa: Cheilostomatida) in the central North Atlantic Azores Archipelago
Source: BMC Ecol Evol. 2022 Nov 4;22:128. doi: 10.1186/s12862-022-02080-z (PMC9635095; doi:10.1186/s12862-022-02080-z)
Supplement: Supplementary file 2 — Supplementary Material 2 [file 12862_2022_2080_MOESM2_ESM.docx]

**Supplementary Information**

**Additional file 2**

**Morphospecies and molecular diversity of ‘lace corals’: the genus *Reteporella* (Bryozoa: Cheilostomatida) in the central North Atlantic Azores Archipelago**

Lara Baptista^1,2,3,4,5 *^, Björn Berning^1,6^, Manuel Curto^5,7^, Andrea Waeschenbach ^8,^ Harald Meimberg^5^, António M. Santos^4,9^, Sérgio P. Ávila^1,2,3,4,10^

^1^ CIBIO, Centro de Investigação em Biodiversidade e Recursos Genéticos, InBIO Laboratório Associado, Pólo dos Açores, 9501-801 Ponta Delgada, Açores, Portugal. ^2^ BIOPOLIS Program in Genomics, Biodiversity and Land Planning, CIBIO, Campus de Vairão, 4485-661 Vairão, Portugal. ^3^ MPB-Marine Palaeontology and Biogeography Lab, Universidade dos Açores, 9501-801 Ponta Delgada, Açores, Portugal. ^4^ Faculdade de Ciências da Universidade do Porto, Rua do Campo Alegre 1021/1055, 4169-007 Porto, Portugal. ^5^ Institute for Integrative Nature Conservation Research, University of Natural Resources and Life Sciences (BOKU), Vienna, Austria. ^6^ Oberösterreichische Landes-Kultur GmbH, Geowissenschaftliche Sammlungen, Leonding 4060, Austria. ^7^ MARE, Marine and Environmental Sciences Centre, Faculdade de Ciências, Universidade de Lisboa, Campo Grande, Lisboa, 1749-016 Portugal. ^8^ Natural History Museum, London, United Kingdom.^9^ CIBIO, Centro de Investigação em Biodiversidade e Recursos Genéticos, InBIO Laboratório Associado, Campus de Vairão, Universidade do Porto, 4485-661 Vairão, Portugal. ^10^ Departamento de Biologia, Faculdade de Ciências e Tecnologia, Universidade dos Açores, 9501-801 Ponta Delgada, Açores, Portugal

*Corresponding author: L. Baptista (laracbaptista@hotmail.com); ORCID: 0000-0002-4429-9855

**Table S2 – Estimates of evolutionary divergence (mean raw p-distances) between Reteporella species and additional phidoloporids.** **a)** mitochondrial COI marker (552 bp); **b)** mitochondrial 16S rRNA marker (332 bp); and **c)** nuclear 28S rRNA marker (427 bp). The analysis, using MEGA v11 [2], was performed after exclusion of positions containing gaps and/or missing data for each sequence pair analysed. Intraspecific mean p-distances were calculated among haplotypes for each species. Hyphen (-) indicates species represented by a single haplotype. Samples R. atlantica (BRY16; Flores) and Reteporella sp. 5 (BRY257; Formigas) are distinguished in a) and b) due to their differentiation when compared to conspecifics. Pairwise comparisons of other phidoloporid genera highlighted in yellow.

| **a)** | R. atlantica | R. atlantica (BRY16) | R. oceanica | R. sp. 5 | R. sp. 6 | R. sp. 7 | R. sp. 7 (BRY257) | R. sp. 1 | R. tristis | R. beaniana | R. sp. 2 | R. sp. 3 | R. cf. grimaldii | R. sp. 4 | R. cf. graeffei | R. tuberosa | P. avicularis | Schizoretepora sp. | Schizotheca sp. | H. novaezelandiae | T. arcuatum | I. yaldwyni | I. violaceum |
| --- | --- | --- | --- | --- | --- | --- | --- | --- | --- | --- | --- | --- | --- | --- | --- | --- | --- | --- | --- | --- | --- | --- | --- |
| R. atlantica | 0.021 |  |  |  |  |  |  |  |  |  |  |  |  |  |  |  |  |  |  |  |  |  |  |
| R. atlantica (BRY16) | 0.033 | - |  |  |  |  |  |  |  |  |  |  |  |  |  |  |  |  |  |  |  |  |  |
| R. oceanica | 0.067 | 0.082 | - |  |  |  |  |  |  |  |  |  |  |  |  |  |  |  |  |  |  |  |  |
| Reteporella sp. 5 | 0.083 | 0.098 | 0.038 | - |  |  |  |  |  |  |  |  |  |  |  |  |  |  |  |  |  |  |  |
| Reteporella sp. 6 | 0.087 | 0.097 | 0.066 | 0.071 | 0.012 |  |  |  |  |  |  |  |  |  |  |  |  |  |  |  |  |  |  |
| Reteporella sp. 7 | 0.070 | 0.078 | 0.083 | 0.083 | 0.057 | 0.017 |  |  |  |  |  |  |  |  |  |  |  |  |  |  |  |  |  |
| Reteporella sp. 7 (BRY257) | 0.089 | 0.101 | 0.089 | 0.091 | 0.063 | 0.034 | - |  |  |  |  |  |  |  |  |  |  |  |  |  |  |  |  |
| Reteporella sp. 1 | 0.179 | 0.183 | 0.178 | 0.183 | 0.185 | 0.187 | 0.194 | - |  |  |  |  |  |  |  |  |  |  |  |  |  |  |  |
| R. tristis | 0.201 | 0.205 | 0.206 | 0.201 | 0.193 | 0.196 | 0.202 | 0.097 | 0.012 |  |  |  |  |  |  |  |  |  |  |  |  |  |  |
| R. beaniana | 0.178 | 0.183 | 0.183 | 0.183 | 0.191 | 0.194 | 0.201 | 0.172 | 0.162 | - |  |  |  |  |  |  |  |  |  |  |  |  |  |
| Reteporella sp. 2 | 0.170 | 0.171 | 0.174 | 0.176 | 0.166 | 0.171 | 0.172 | 0.152 | 0.168 | 0.132 | - |  |  |  |  |  |  |  |  |  |  |  |  |
| Reteporella sp. 3 | 0.175 | 0.174 | 0.190 | 0.181 | 0.164 | 0.180 | 0.181 | 0.156 | 0.176 | 0.147 | 0.062 | 0.005 |  |  |  |  |  |  |  |  |  |  |  |
| R. cf. grimaldii | 0.172 | 0.172 | 0.176 | 0.170 | 0.159 | 0.180 | 0.179 | 0.165 | 0.180 | 0.134 | 0.042 | 0.061 | - |  |  |  |  |  |  |  |  |  |  |
| Reteporella sp. 4 | 0.138 | 0.147 | 0.132 | 0.130 | 0.134 | 0.143 | 0.138 | 0.179 | 0.195 | 0.190 | 0.182 | 0.181 | 0.175 |  |  |  |  |  |  |  |  |  |  |
| R. cf. graeffei | 0.135 | 0.143 | 0.129 | 0.132 | 0.136 | 0.142 | 0.138 | 0.176 | 0.196 | 0.192 | 0.185 | 0.187 | 0.181 | 0.002 | - |  |  |  |  |  |  |  |  |
| R. tuberosa | 0.222 | 0.228 | 0.232 | 0.234 | 0.226 | 0.216 | 0.225 | 0.210 | 0.224 | 0.236 | 0.205 | 0.216 | 0.199 | 0.222 | 0.221 | - |  |  |  |  |  |  |  |
| Phidolopora avicularis | 0.209 | 0.203 | 0.228 | 0.225 | 0.218 | 0.235 | 0.241 | 0.219 | 0.232 | 0.219 | 0.210 | 0.210 | 0.201 | 0.238 | 0.236 | 0.197 | - |  |  |  |  |  |  |
| Schizoretepora sp. | 0.240 | 0.232 | 0.252 | 0.25 | 0.243 | 0.238 | 0.246 | 0.241 | 0.235 | 0.232 | 0.234 | 0.239 | 0.234 | 0.263 | 0.259 | 0.216 | 0.170 | - |  |  |  |  |  |
| Schizotheca sp. | 0.203 | 0.205 | 0.219 | 0.208 | 0.209 | 0.218 | 0.23 | 0.210 | 0.214 | 0.208 | 0.223 | 0.231 | 0.221 | 0.233 | 0.230 | 0.205 | 0.194 | 0.201 | - |  |  |  |  |
| Hippellozoon novaezelandiae | 0.215 | 0.216 | 0.241 | 0.23 | 0.230 | 0.218 | 0.23 | 0.205 | 0.231 | 0.212 | 0.212 | 0.226 | 0.210 | 0.242 | 0.245 | 0.188 | 0.181 | 0.174 | 0.194 | - |  |  |  |
| Triphyllozoon arcuatum | 0.255 | 0.257 | 0.259 | 0.255 | 0.241 | 0.246 | 0.252 | 0.236 | 0.244 | 0.243 | 0.223 | 0.231 | 0.226 | 0.263 | 0.259 | 0.207 | 0.228 | 0.25 | 0.23 | 0.228 | - |  |  |
| Iodictyum yaldwyni | 0.252 | 0.261 | 0.255 | 0.257 | 0.242 | 0.242 | 0.255 | 0.221 | 0.224 | 0.23 | 0.228 | 0.240 | 0.230 | 0.261 | 0.248 | 0.194 | 0.236 | 0.246 | 0.234 | 0.208 | 0.217 | - |  |
| Iodictyum violaceum | 0.241 | 0.241 | 0.252 | 0.261 | 0.242 | 0.252 | 0.257 | 0.234 | 0.253 | 0.228 | 0.210 | 0.216 | 0.219 | 0.250 | 0.261 | 0.217 | 0.219 | 0.219 | 0.221 | 0.187 | 0.225 | 0.178 | - |

| **b)** | R. atlantica | R. atlantica (BRY16) | R. sp. 5 | R. sp. 6 | R. sp. 7 | R. sp. 7 (BRY257) | R. sp. 1 | R. tristis | R. sp. 2 | R. sp. 3 | R. cf. grimaldii | R. cf. graeffei | R. tuberosa | I. yaldwyni | H. novaezelandiae | T. arcuatum | P. avicularis |
| --- | --- | --- | --- | --- | --- | --- | --- | --- | --- | --- | --- | --- | --- | --- | --- | --- | --- |
| R. atlantica | 0.002 |  |  |  |  |  |  |  |  |  |  |  |  |  |  |  |  |
| R. atlantica (BRY16) | 0.003 | - |  |  |  |  |  |  |  |  |  |  |  |  |  |  |  |
| Reteporella sp. 5 | 0.022 | 0.020 | - |  |  |  |  |  |  |  |  |  |  |  |  |  |  |
| Reteporella sp. 6 | 0.013 | 0.011 | 0.010 | 0.005 |  |  |  |  |  |  |  |  |  |  |  |  |  |
| Reteporella sp. 7 | 0.016 | 0.010 | 0.008 | 0.007 | 0.002 |  |  |  |  |  |  |  |  |  |  |  |  |
| Reteporella sp. 7 (BRY257) | 0.012 | 0.013 | 0.009 | 0.005 | 0.003 | - |  |  |  |  |  |  |  |  |  |  |  |
| Reteporella sp. 1 | 0.085 | 0.083 | 0.085 | 0.081 | 0.081 | 0.080 | - |  |  |  |  |  |  |  |  |  |  |
| R. tristis | 0.085 | 0.083 | 0.080 | 0.080 | 0.077 | 0.080 | 0.026 | 0 |  |  |  |  |  |  |  |  |  |
| Reteporella sp. 2 | 0.043 | 0.036 | 0.036 | 0.040 | 0.039 | 0.039 | 0.067 | 0.063 | - |  |  |  |  |  |  |  |  |
| Reteporella sp. 3 | 0.044 | 0.038 | 0.037 | 0.041 | 0.040 | 0.040 | 0.069 | 0.061 | 0.004 | 0.002 |  |  |  |  |  |  |  |
| R. cf. grimaldii | 0.045 | 0.039 | 0.031 | 0.035 | 0.034 | 0.033 | 0.070 | 0.065 | 0.007 | 0.008 | - |  |  |  |  |  |  |
| R. cf. graeffei | 0.036 | 0.035 | 0.023 | 0.027 | 0.027 | 0.028 | 0.096 | 0.096 | 0.056 | 0.057 | 0.050 | - |  |  |  |  |  |
| R. tuberosa | 0.198 | 0.198 | 0.198 | 0.197 | 0.192 | 0.196 | 0.208 | 0.213 | 0.197 | 0.198 | 0.193 | 0.196 | - |  |  |  |  |
| Iodictyum yaldwyni | 0.177 | 0.177 | 0.172 | 0.171 | 0.166 | 0.169 | 0.184 | 0.184 | 0.175 | 0.173 | 0.169 | 0.169 | 0.111 | - |  |  |  |
| Hippellozoon novaezelandiae | 0.181 | 0.179 | 0.175 | 0.176 | 0.169 | 0.173 | 0.178 | 0.183 | 0.174 | 0.178 | 0.172 | 0.180 | 0.094 | 0.087 | - |  |  |
| Triphyllozoon arcuatum | 0.200 | 0.197 | 0.195 | 0.195 | 0.189 | 0.192 | 0.207 | 0.200 | 0.205 | 0.204 | 0.201 | 0.195 | 0.121 | 0.132 | 0.123 | - |  |
| Phidolopora avicularis | 0.187 | 0.181 | 0.182 | 0.181 | 0.176 | 0.180 | 0.190 | 0.190 | 0.193 | 0.192 | 0.189 | 0.180 | 0.109 | 0.082 | 0.055 | 0.127 | - |

| **c)** | R. atlantica | R. oceanica | R. sp. 3 | R. sp. 4 | R. sp. 5 | R. sp. 1 | R. tristis | R. beaniana | R. sp. 2 | R. sp. 3 | R. cf. grimaldii | R. cf. graeffei | P. avicularis | H. novaezelandiae | I. violaceum | T. arcuatum |
| --- | --- | --- | --- | --- | --- | --- | --- | --- | --- | --- | --- | --- | --- | --- | --- | --- |
| R. atlantica | 0 |  |  |  |  |  |  |  |  |  |  |  |  |  |  |  |
| R. oceanica | 0.003 | - |  |  |  |  |  |  |  |  |  |  |  |  |  |  |
| Reteporella sp. 5 | 0.003 | 0 | - |  |  |  |  |  |  |  |  |  |  |  |  |  |
| Reteporella sp. 6 | 0.003 | 0 | 0 | 0 |  |  |  |  |  |  |  |  |  |  |  |  |
| Reteporella sp. 7 | 0.003 | 0 | 0 | 0 | 0 |  |  |  |  |  |  |  |  |  |  |  |
| Reteporella sp. 1 | 0.006 | 0.003 | 0.003 | 0.003 | 0.003 | - |  |  |  |  |  |  |  |  |  |  |
| R. tristis | 0.006 | 0.003 | 0.003 | 0.003 | 0.003 | 0.003 | 0 |  |  |  |  |  |  |  |  |  |
| R. beaniana | 0.006 | 0.003 | 0.003 | 0.003 | 0.003 | 0.006 | 0.006 | - |  |  |  |  |  |  |  |  |
| Reteporella sp. 2 | 0.012 | 0.015 | 0.015 | 0.015 | 0.015 | 0.015 | 0.012 | 0.012 | - |  |  |  |  |  |  |  |
| Reteporella sp. 3 | 0.012 | 0.015 | 0.015 | 0.015 | 0.015 | 0.015 | 0.012 | 0.012 | 0 | - |  |  |  |  |  |  |
| R. cf. grimaldii | 0.012 | 0.015 | 0.015 | 0.015 | 0.015 | 0.015 | 0.012 | 0.012 | 0 | 0 | - |  |  |  |  |  |
| R. cf. graeffei | 0.003 | 0 | 0 | 0 | 0 | 0.003 | 0.003 | 0.003 | 0.015 | 0.015 | 0.015 | - |  |  |  |  |
| Phidolopora avicularis | 0.066 | 0.063 | 0.063 | 0.063 | 0.063 | 0.060 | 0.063 | 0.030 | 0.060 | 0.060 | 0.060 | 0.063 | - |  |  |  |
| Hippellozoon novaezelandiae | 0.030 | 0.033 | 0.033 | 0.033 | 0.033 | 0.030 | 0.033 | 0.036 | 0.033 | 0.033 | 0.033 | 0.033 | 0.069 | - |  |  |
| Iodictyum violaceum | 0.027 | 0.030 | 0.030 | 0.030 | 0.030 | 0.033 | 0.033 | 0.063 | 0.027 | 0.027 | 0.027 | 0.030 | 0.073 | 0.048 | - |  |
| Triphyllozoon arcuatum | 0.018 | 0.021 | 0.021 | 0.021 | 0.021 | 0.021 | 0.018 | 0.024 | 0.012 | 0.012 | 0.012 | 0.021 | 0.054 | 0.039 | 0.036 | - |


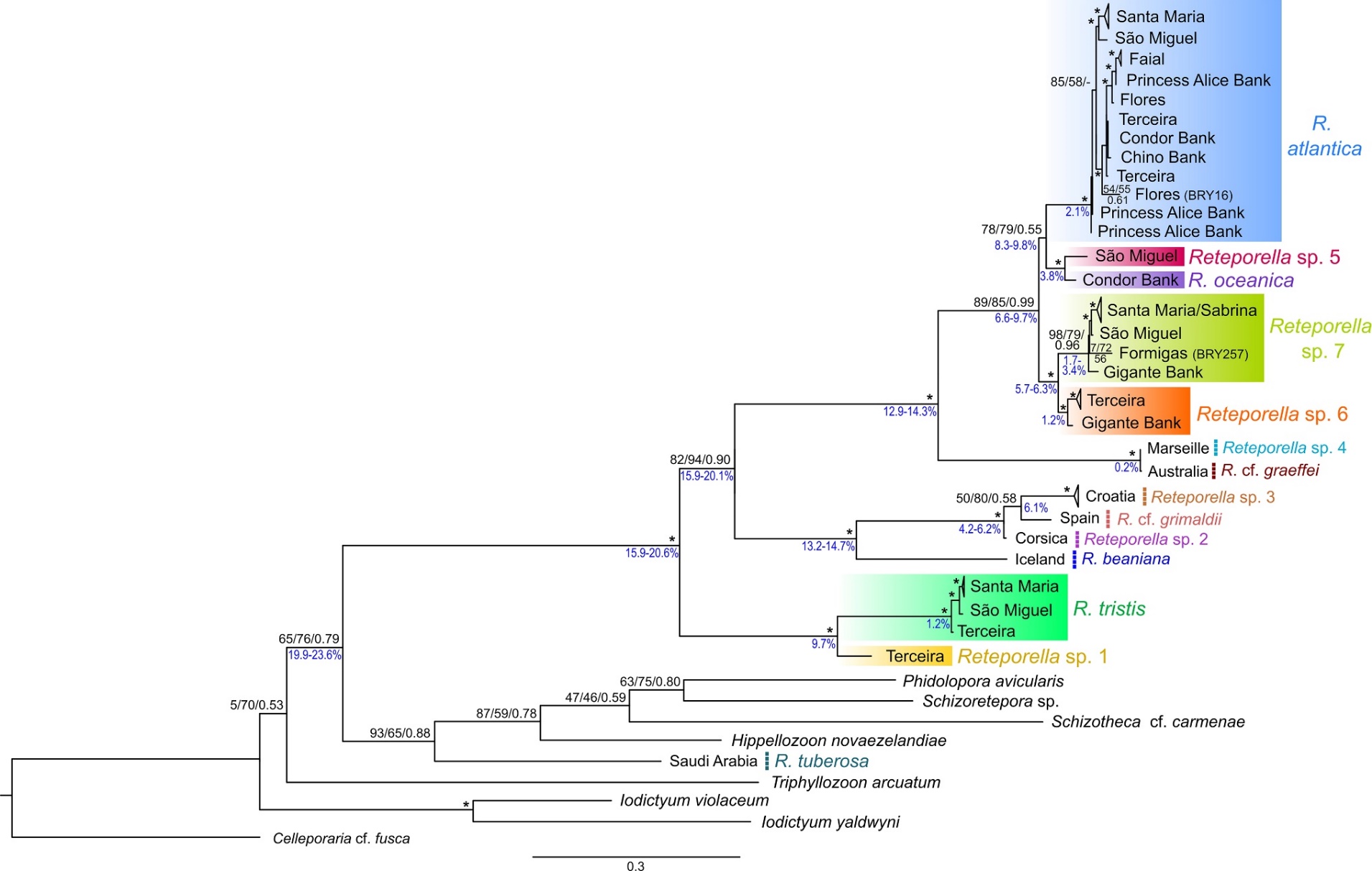


**Figure S1 – Maximum likelihood analysis of the COI data.** Constructed using the W-IQ-Tree web server [3] under the GTR+I (1^st^ codon position), F81+I (2^nd^ codon position) and GTR+G (3^rd^ codon position) models of nucleotide evolution. Values at the nodes correspond to branch test (SH-aLTR) values, ultrafast bootstrap support (UFboot) values, and posterior probabilities (PP), inferred with W-IQ-Tree and MrBayes v3.2.7 software [4], respectively. Hyphen (-) indicates nodes absent in one of the phylogenetic reconstructions; asterisk (*) indicates nodes supported by both ML and BI analyses (SH-aLTR>= 80%, UFboot >= 95%, PP >= 95%). Mean divergence levels among *Reteporella* terminals, as estimated with MEGA11 [2], are depicted at the nodes in blue. Known and putative new Azorean *Reteporella* species are indicated with different coloured shading. Non-Azorean species are indicated by dashed coloured lines. Other phidoloporids included in the analyses are given in normal black font. Geographic origins of *Reteporella* samples are given as terminal labels. The scale bar represents substitutions per site.


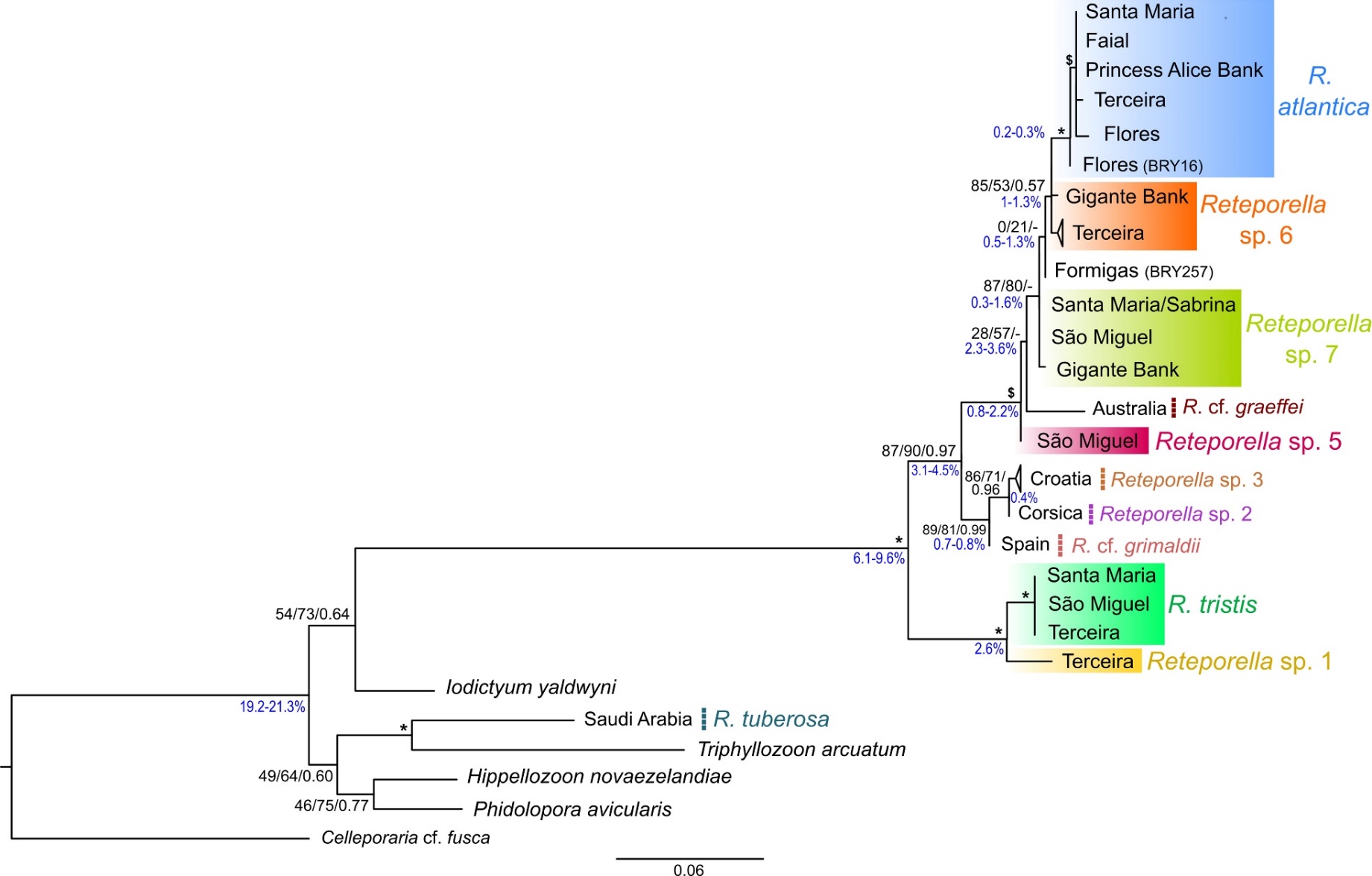


**Figure S2 – Maximum likelihood analysis of the 16S rRNA data**. Constructed using the W-IQ-Tree web server [3] under the GTR+I+G model of nucleotide evolution. Values at the nodes correspond to branch test (SH-aLTR) values, ultrafast bootstrap support (UFboot) values, and posterior probabilities (PP), inferred with W-IQ-Tree and MrBayes v3.2.7 software [4], respectively. Hyphen (-) indicates nodes absent in one of the phylogenetic reconstructions; asterisk (*) indicates nodes supported by both ML and BI analyses (SH-aLTR>= 80%, UFboot >= 95%, PP >= 95%); dollar sign ($) indicates nodes supported only by ML analyses (SH-aLTR>= 80%, UFboot >= 95%). Mean divergence levels among *Reteporella* terminals, as estimated with MEGA11 [2], are depicted at the nodes in blue. Known and putative new Azorean *Reteporella* species are indicated with different coloured shading. Non-Azorean *Reteporella* species are indicated by dashed coloured lines. Geographical origins of the *Reteporella* samples are given as terminal labels. Other phidoloporids included in the analyses are given in normal black font. Geographic origins of *Reteporella* samples are given as terminal labels. The scale bar represents substitutions per site.


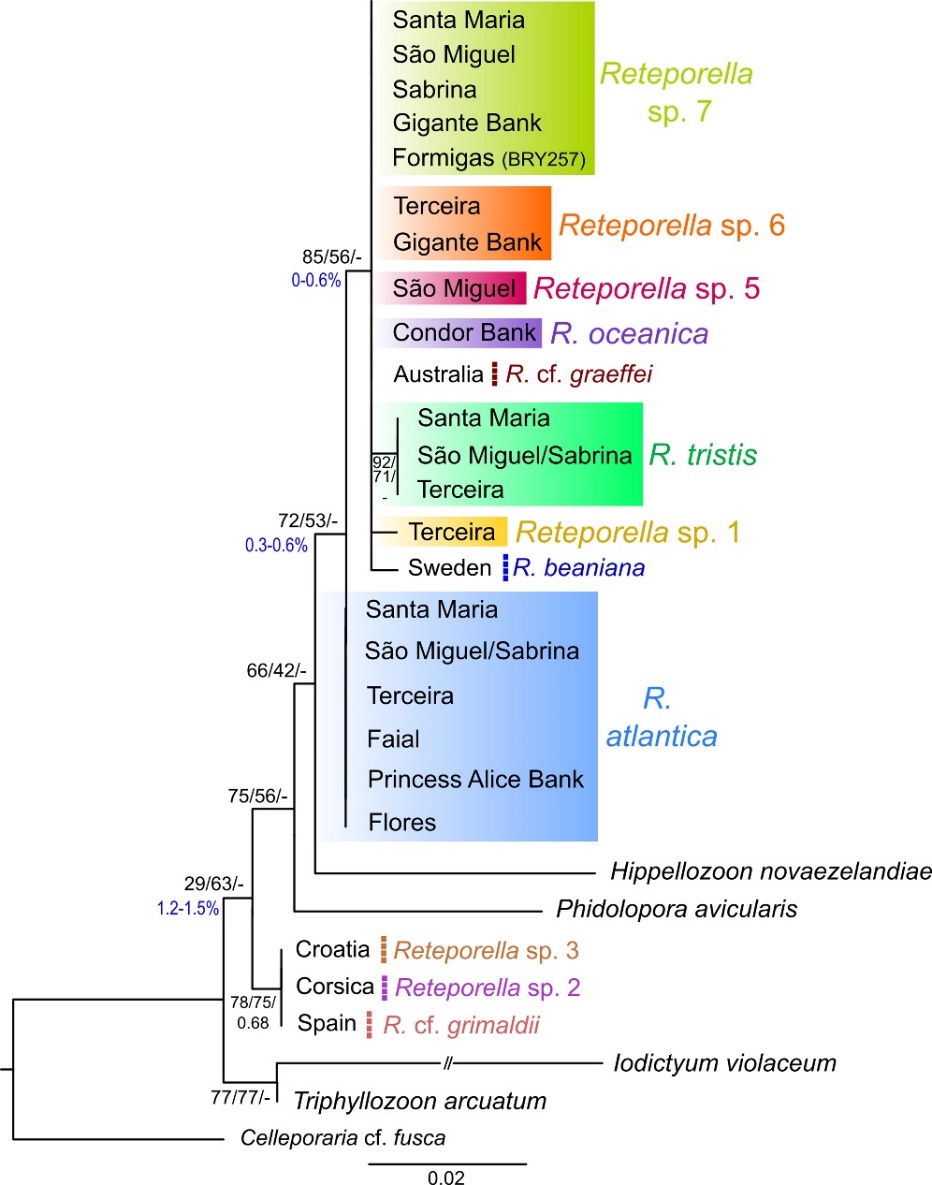


**Figure S3 – Maximum likelihood analysis of the 28S rRNA data.** Constructed using the W-IQ-Tree web server [3] under the GTR+I+G model of nucleotide evolution. Values at the nodes correspond to branch test (SH-aLTR) values, ultrafast bootstrap support (UFboot) values, and posterior probabilities (PP), inferred with W-IQ-Tree and MrBayes v3.2.7 software [4], respectively. Hyphen (-) indicates nodes absent in one of the phylogenetic reconstructions; asterisk (*) indicates nodes supported by both ML and BI analyses (SH-aLTR>= 80%, UFboot >= 95%, PP >= 95%). Mean divergence levels among *Reteporella* terminals, as estimated with MEGA11 [2], are depicted at the nodes in blue. Known and putative new Azorean *Reteporella* species are indicated with different coloured shading. Non-Azorean *Reteporella* species are indicated by dashed coloured lines. Geographical origins of the *Reteporella* samples are given as terminal labels. Other phidoloporids included in the analyses are given in normal black font. Geographic origins of *Reteporella* samples are given as terminal labels. The scale bar represents substitutions per site.

**References**

1. Wisshak M, Berning B, Jakobsen J, Freiwald A. Temperate carbonate production: biodiversity of calcareous epiliths from intertidal to bathyal depths (Azores). Mar Biodivers. 2015;45:87–112. <https://doi.org/10.1007/s12526-014-0231-6>.

2. Tamura K, Stecher G, Kumar S. MEGA11: Molecular Evolutionary Genetics Analysis Version 11. Mol Biol Evol. 2021;38:3022–7. <https://doi.org/10.1093/molbev/msab120>.

3. Trifinopoulos J, Nguyen LT, von Haeseler A, Minh BQ. W-IQ-TREE: a fast online phylogenetic tool for maximum likelihood analysis. Nucleic Acids Res. 2016;44:W232-5. <https://doi.org/10.1093/nar/gkw256>.

4. Ronquist F, Teslenko M, Van Der Mark P, Ayres DL, Darling A, Höhna S, et al. MBayes 3.2: Efficient Bayesian phylogenetic inference and model choice across a large model space. Syst Biol. 2012;61:539–42. <https://doi.org/10.1093/sysbio/sys029>.
